# Exploring the Potential of Lactic Acid Fermentation for the Recovery of Exhausted Vanilla Beans

**DOI:** 10.3389/fnut.2022.858716

**Published:** 2022-05-19

**Authors:** Jasmine Hadj Saadoun, Alessia Levante, Antonio Ferrillo, Francesca Trapani, Valentina Bernini, Gianni Galaverna, Erasmo Neviani, Camilla Lazzi

**Affiliations:** ^1^Department of Food and Drug, University of Parma, Parma, Italy; ^2^Enrico Giotti S.p.A. a Subsidiary of McCormick & Company, Inc, Firenze, Italy; ^3^Interdepartmental Center, SITEIA.PARMA—Centro Interdipartimentale sulla Sicurezza, Tecnologie e Innovazione Agroalimentare, University of Parma, Parma, Italy

**Keywords:** fermentation, by-product, vanilla beans, aromatic compounds, vanillin, Response Surface Methodology

## Abstract

The market value of vanilla is constantly growing, as it is the aroma most appreciated by consumers worldwide. The key component of the aroma of vanilla beans is vanillin, which can be directly extracted from the plant, produced by chemical synthesis, or by bioconversion of natural precursors. Due to the increasing consumers' demand for products labeled as “natural,” extraction from vanilla pods results in a more valuable aroma source. Once the extraction is completed, what remains are the exhausted beans that still contain small seeds and other compounds, including varying amounts of vanillin trapped in the cellular structures of the plant. The application of fermentation of exhausted vanilla beans is proposed here as a strategy to recover “natural” vanillin and other valuable aroma compounds as a result of the metabolic conversion by lactic acid bacteria (LAB). The aim of this study was to verify the fermentability of exhausted vanilla beans by-products for their valorization, allowing the recovery of high-value molecules or new applications in food products. Design of Experiment (DoE) was used to screen a library of LAB strains to identify the best condition of fermentation in response to varying cultivation conditions. A comparison between mono and co-culture of LAB was assessed. Moreover, sensory panel tests and the evaluation of the aromatic components by Solid Phase Micro Extraction-Gas Chromatography-Mass Spectrometry analysis were carried out to better understand the modification of the aroma profile after fermentation. Fermentation with LAB changed the volatile profile and sensory characteristics of the exhausted vanilla beans and represents a promising method for the valorization of these by-products.

## Introduction

Vanilla (*Vanilla planifolia*) is a tropical orchid famous worldwide for its aroma. The market value of vanilla is constantly growing as it is the most appreciated aroma by consumers and is therefore used in food preparation as desserts, ice creams and soft drinks, and spirits (1). In 2017, the global vanilla market size was 65 million US$ but it is expected to grow in the next years reaching 100 million US$ in 2025 (2).

Among the main components of the aroma, the most important and abundant in vanilla beans is vanillin. Vanillin can be directly extracted from the plant and can be produced by chemical synthesis or by bioconversion of ferulic acid (3–5). To meet the growing global demand, the chemical synthesis of vanillin has become a major source, using guaiacol and lignin as starting materials. This choice has disadvantages due to the large quantities of pollutants, which make it an unsustainable environmental technique (6). Moreover, in recent years consumers have become more aware of products labeled as “natural,” thus favoring those products using aroma extracted directly from the vanilla beans. Once the beans have been harvested, they must ripen and begin the curing process that consists of different steps in which vanilla beans develop their typical aroma due to enzymatic processes mainly linked to the action of β-glucosidases present in the vegetal tissue (7). The extraction consists in leaving the pods in a solution of water and ethanol, and a vacuum can be applied to enhance the process (8). Once the extraction is complete, what remains are the exhausted beans that “still contain small seeds. The small vanilla seeds present in the exhausted pods still retain a certain economic value because they are added to food products to accomplish consumers” demand for increased naturalness of foods. For this reason, a further separation process can be performed to recover the seeds, leaving a residual by-product without seeds, but still characterized by other volatile compounds as well as varying amounts of vanillin, trapped in the cellular structures of the plant (9). Thus, the two by-products of vanillin extraction, i.e., vanilla pods with seeds and without seeds, represent a valuable resource for aroma production companies, with potential for repurposing and valorization.

Another technique that can be used to produce “natural” vanillin is fermentation. This technique allows the conversion of natural substances such as lignin, ferulic acid, and eugenol present in the beans of vanilla thanks to the enzyme portfolio of microorganisms (10). Recent studies reported the conversion into vanillin by microorganisms using agro-industrial waste like cereal bran, sugar beet pulp, or rice bran oil (11, 12).

In recent years, the recovery of valuable compounds from plant-based byproducts using bioconversions like enzymatic treatment or fermentation has been explored by various authors, as reviewed by Tlais et al. (13). Among the possible strategies, solid-state fermentation (SSF) has been widely used for the recovery of bioactive molecules from agro-industrial by-products using Lactic Acid Bacteria (LAB) (14, 15). LAB consists of a heterogeneous group of Gram-positive bacteria recognized as GRAS, widely used in the food industry as starters as they can grow on many different carbon sources and have a good tolerance to environmental stresses (16).

Recently, this approach had been applied to a variety of plant-based byproducts, proving the effectiveness of SSF in producing aromatic compounds from melon and apple by-products (17, 18). Volatile compounds are not the only outcomes of fermentation, indeed often a change in the phenolic profile of the by-products is obtained (19, 20), an improvement of their nutritional value (21), or the production of bioactive compounds such as antimicrobials (22). Besides, some LAB is capable to develop in substrates characterized by a high phenolic content, such as elderberry juice. In this substrate, Ricci et al. (23) have shown how the cultivation of isolates of dairy origin produces different volatile compounds compared to those isolated from vegetable matrices. Furthermore, another study has observed that the effect of LAB on the substrate could be linked not only to a growth phenotype but also in cases where no growth or cell lysis occurred, changes were observed in terms of volatile profile and phenolic content (24). Adaptation of the strains to the substrate is a complex process and shows a high variability even among isolates of the same species coming from different niches (25).

Considering the limited availability of vanilla pods and the environmental costs for cultivation, transport, and processing, we propose fermentation with LAB of vanilla by-products for their valorization. Fermentation of exhausted vanilla beans could be a valuable strategy for the recovery of part of the valuable aroma compounds which are not primarily extracted, or the fermented substrate can be used as a novel ingredient for application in sweets and confectionery products. For the fermentation study, the suitability of the two different by-products, with and without seeds, was evaluated to study if both are suitable for this application and to determine if the seeds' presence in the fermented substrate enhances the overall flavor performance. To the best of our knowledge, no studies are present in the literature regarding the fermentation and characterization of sensory and volatile profiles of vanilla by-products.

In this study, the Design of Experiments (DoE) was evaluated to assess the effect of different parameters on the development of 8 different species of LAB strains in single culture and to identify conditions for the setup of co-culture experiments. Based on fermentation results, sensory analysis was performed to detect changes in the taste for further food applications. On the same samples, Solid Phase Micro Extraction-Gas Chromatography-Mass Spectrometry (SPME-GC/MS) was applied to measure changes in the volatile compounds' concentration, for aroma extraction purposes.

## Materials and Methods

### Substrates

**Figure 1:**
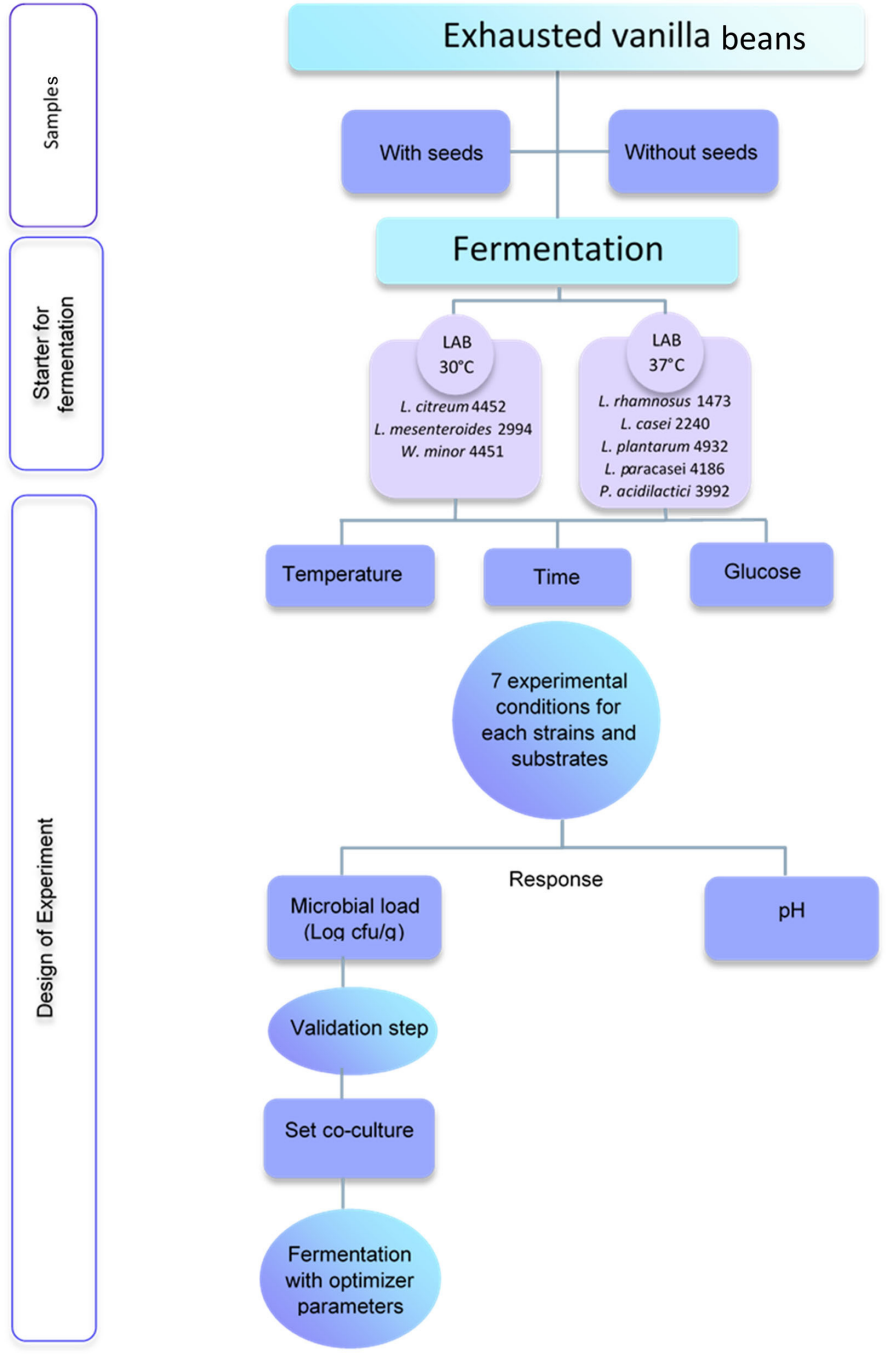
Experimental procedure for fermentation of exhausted vanilla beans.

The substrate understudy was exhausted vanilla beans after the vanillin extraction. Figure 1 shows the experimental procedure. Two different samples were taken into consideration: vanilla beans with seeds (S) and without seeds (NS). Both substrates were autoclaved prior to fermentation with the selected strains. Samples were provided by the Giotti-McCormick company (Firenze, Italy).

### Bacterial Strains

For this study, eight bacterial strains isolated from various food matrices and belonging to different LAB species were used for fermentation. Bacterial strains with an optimal growth temperature of 30°C are *Leuconostoc citreum* 4452 (Sour dough), *Leuconostoc mesenteroides* 2194 (Grana Padano cheese), *Weissella minor* 4451 (Sour dough), while strains with an optimal growth temperature of 37°C are *Lacticaseibacillus rhamnosus* 1473 (Parmigiano Reggiano cheese), *Lacticaseibacillus paracasei* 4186 (Pecorino toscano cheese), *Lacticaseibacillus casei* 2240 (Parmigiano Reggiano cheese), *Lactiplantibacillus plantarum* 4932 (Minas cheese), *Pediococcus acidilactici* 3992 (Grana Padano cheese). All the strains belong to the University of Parma Culture Collection (UPCC) of the Department of Food and Drugs.

The bacterial stock cultures were maintained as frozen at −80°C in De Man Rogosa and Sharpe (MRS) broth (Oxoid, Basingstoke, UK) added with 12.5% glycerol (v/v).

Before fermentation, the strain was transferred twice in MRS broth (3% v/v) and incubated for 24 h at the optimal temperature. Afterward, 200 μl of culture broth was inoculated in 6 ml of MRS broth and incubated at 30–37 C for 15 h to obtain a cell concentration of 9 Log cfu/ml. Bacterial culture was centrifuged (10,000 rpm, 10 min, 4°C), washed twice in Ringer solution (VWR, UK), and suspended in sterile bidistilled water. Before the inoculum in the samples, substrates were sterilized in an autoclave at 121°C for 21 min. Each culture was then inoculated into 10 g of vanilla beans to reach 7 Log cfu/g. Co-cultures were made using strains with the same optimal growth temperatures and were obtained by mixing single revitalized strains in equal volume and further diluting the mixture to reach 7 Log CFU/g in the exhausted vanilla beans.

### Experimental Design of Fermentation

Experiments were designed around a mathematical model using the software MODDE Pro version 12.0.1 (MKS Umetrics, Umeå, Sweden). The model was designed around three quantitative factors: temperature, time of fermentation, and the concentration of glucose added to the substrates. The temperature for the experimental conditions ranged between 32 and 42°C for strains with an optimal growth temperature of 37°C, while for strains whose optimal growth temperature was 30°C the experimental temperature ranged between 25 and 35°C. The time set for the fermentation ranged between 30 and 120 h, while the added glucose varied between no addition (0%) and 5% (w/w).

The measured responses were the microbial load (Log cfu/g) and the variation of pH. The Response Surface Methodology (RSM) model was drawn up with a fractional factorial design. The fractional factorial design is a screening model often applied to overview a biological phenomenon, at the same time allowing the definition of optimum value (26).

Each strain was used for the fermentation of the substrate(s) under the conditions defined by the DoE, following the matrix generated from the software with different temperatures of incubation, according to the optimal ones for each strain. The microbial load was evaluated at the end of the incubation time, through a plate count on MRS agar. Plates were incubated at 30–37°C for 48 h.

The pH measurement was performed in the sample diluted 1:10 in distilled water and the analysis was conducted using a pH meter (Mettler Toledo, Columbus, Ohio, US). The pH was evaluated in the unfermented sample (control) and after fermentation.

Once the data were obtained for each strain, they were used to build a model, producing a response surface that gave us indications for obtaining the maximum response, i.e., the highest microbial load. According to the indications produced using the model in combination with a response optimization algorithm, growth conditions were postulated for the set-up of microbial co-cultures, as described in paragraph 2.2.

### Sensory Analysis

The fermented samples were evaluated through a sensory analysis to understand if the fermentation can enhance the sweet taste, for future identification of natural sweeteners deriving from this process, and to identify aromatic notes of interest that can be characterized and extracted in a subsequent study.

**Figure 2:**
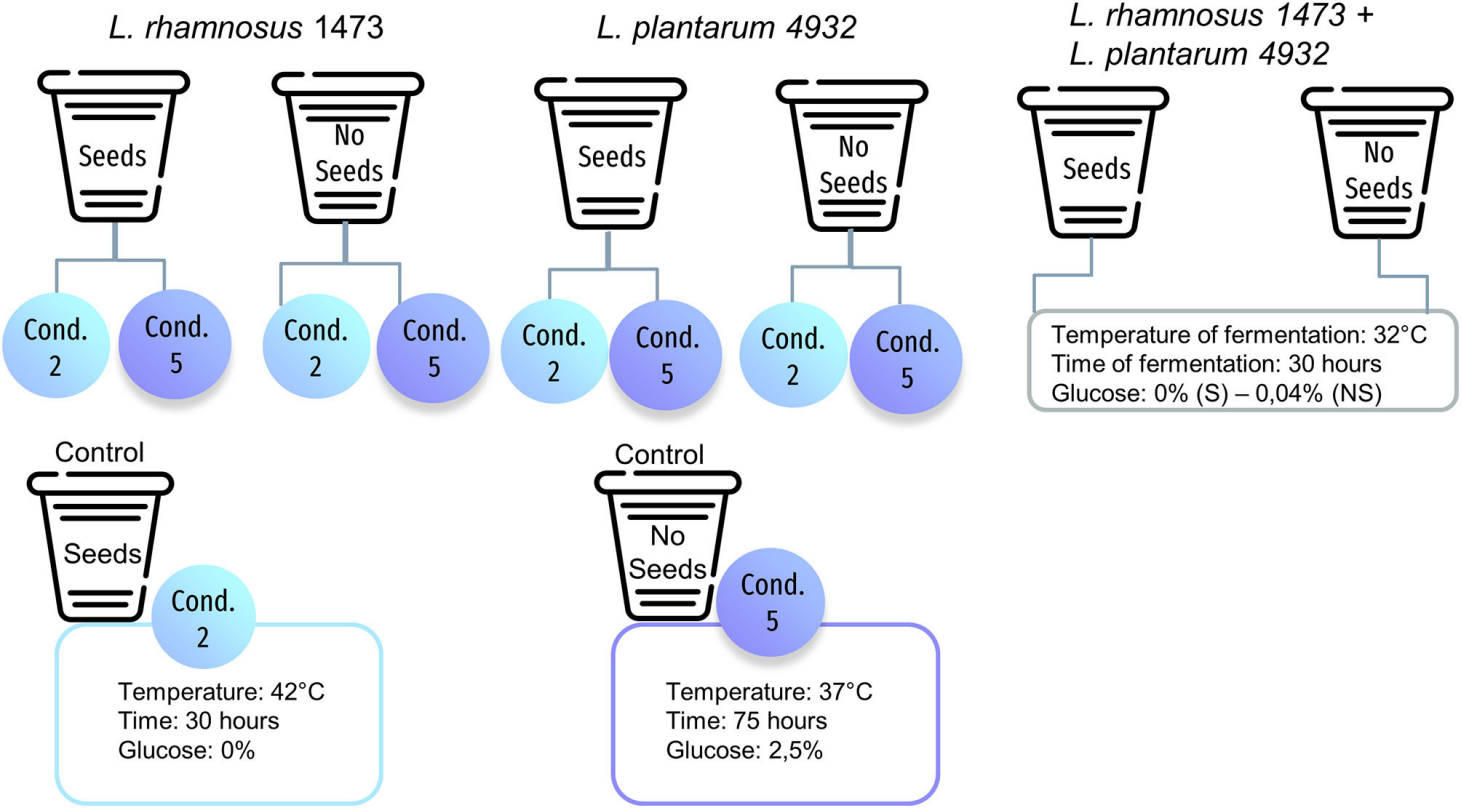
Example of experimental conditions used for sensory analysis for strains with the optimal growth temperature of 37°C. S is the sample with seeds; NS is the sample without seeds.

The samples (Figure 2) were evaluated by a panel of 12 trained sensory assessors of Giotti-McCormick Company, in two replicates for their aroma (AR), flavor (FL), and aftertaste (AT). A Preference Maps test was carried out using the Sorting techniques (27).

The order of sample presentation was randomized across assessors. Results were collected *via* the Compusense data capture system and were analyzed with XLSTAT software (Addinsoft, Paris, France) using ANOVA (*P* < 0.05).

The panelists received seven different samples of vanilla beans that differed in strains used and experimental conditions of fermentation. For this evaluation, we chose the following samples: for each monoculture, we selected condition n. 2, with no addition of glucose and fewer hours of fermentation; condition n. 5, performed at the optimal growth temperature for each strain, with the addition of sugar and longer fermentation time. For the co-culture, we selected the optimal condition generated by DoE prediction. Finally, two control samples (unfermented) were prepared for conditions n.2 and n.5. This part of the study is focused on evaluating whether there are sensory differences in samples where a different increase in microbial load was recorded, and assessing the effect of different growth conditions which were tested as follows: the co-culture, the condition n.2 which is the most far from the optimal one, and the condition n.5, which reflects the optimal temperature for the growth of the strain. The choice of the strains was made according to data obtained during fermentation.

Samples were tested in a solution of water and glucose (3°Bx), presented with a three-digit numerical code in sequential and balanced order for all participants. The “Compusense” Sensory Analysis software was used to carry out the test, the Principal Component Analysis (PCA) and Quantitative Descriptive Analysis (QDA). The analysis was conducted both for vanilla beans with seeds (S) and without seeds (NS).

### Characterization of the Volatile Fraction

Volatile compounds produced by inoculated bacteria were analyzed using a gas chromatography device coupled with a mass spectrometer. Briefly, 1 g of vanilla sample was weighed into a 20 ml vial, with 3.80 ml of distilled water, 1.60 g of NaCl, and 0p.01 g of Ethyl nonanoate standard. The vial was heated at 40 C for 50 min to equilibrate the system. The SPME fiber, 85 μm carboxen/polydimethylsiloxane Stable Flex™ (Supelco), was inserted through the septum and exposed in the headspace of the vial for 25 min, to allow absorption of the volatile compounds onto the SPME fiber. The SPME fiber was then introduced into the injector port of the gas chromatograph for 5 min in splitless mode, set at 280°C, to desorb the volatile compounds. The desorbed components were analyzed on a capillary column. Compounds identification was based on a comparison of retention indices (RI), and mass spectra. GC data were analyzed by PCA using the package *factoextra* on R (R version 4.0.5).

## Results

### LAB Growth Assessment in Exhausted Vanilla Beans Without Seeds (NS) Using DoE

To identify the optimal fermentation parameters of vanilla beans NS, the by-product of vanillin extraction with the least value, seven experimental conditions were assessed for each strain, combining the time and temperature of fermentation, and the addition of glucose, for a total of 56 experiments. Experiments were distinguished between strains with optimum growth temperature at 37°C (Table 1) and strain with an optimum at 30°C (Table 2). Experimental conditions n. 5, 6, and 7 for both tables are biological replicates to account for natural variance and represent the central point of the experimental model.

**Table 1:** Experimental condition for microorganisms with an optimum at 37°C in vanilla without seeds.

				***Lacticaseibacillus*** ***rhamnosus*** **1473**		***Lacticaseibacillus paracasei*** **4186**		***Lacticaseibacillus casei*** **2240**		***Lactiplantibacillus plantarum*** **4932**		***Pediococcus acidilactici*** **3992**	
**Exp n**.	**Tem. (°C)**	**Time (h)**	**Glucose (%)**	**pH**	**Log (cfu/g)**	**pH**	**Log (cfu/g)**	**pH**	**Log (cfu/g)**	**pH**	**Log (cfu/g)**	**pH**	**Log (cfu/g)**
1	32	30	5	6.47	7.74	6.27	7.69	6.04	8.18	5.90	7.30	6.37	7.24
2	42	30	0	4.93	7.38	4.97	7.01	4.67	8.04	4.97	7.26	4.95	5.41
3	32	120	0	6.56	7.22	5.51	7.36	5.01	7.06	5.04	6.31	5.48	6.56
4	42	120	5	6.57	6.10	6.31	3.00	5.52	4.18	6.64	4.00	6.10	4.88
5	37	75	2.5	5.01	8.22	6.66	3.00	4.90	8.33	6.77	4.00	6.37	6.24
6	37	75	2.5	5.10	8.33	6.64	3.00	4.97	8.26	6.73	4.00	6.47	6.06
7	37	75	2.5	5.14	8.29	6.60	3.00	4.88	8.24	6.80	4.00	6.53	6.33

*pH and cell densities (Log cfu/g) were measured at the end of the fermentation*.

**Table 2:** Experimental condition for microorganisms with an optimum at 30°C in vanilla without seeds.

				***Leuconostoc citreum*** **4452**		***Leuconostoc mesenteroides*** **2194**		***Weissella minor*** **4451**	
**Exp n**.	**Tem. (°C)**	**Time (h)**	**Glucose (%)**	**pH**	**Log (cfu/g)**	**pH**	**Log (cfu/g)**	**pH**	**Log (cfu/g)**
1	25	30	5	6.91	6.80	6.73	6.30	6.69	7.42
2	35	30	0	6.98	6.15	6.78	5.09	6.75	7.05
3	25	120	0	6.99	7.11	6.95	6.78	6.92	7.14
4	35	120	5	6.90	3.00	6.95	3.00	6.88	4.16
5	30	75	2.5	6.77	3.00	6.80	6.27	6.71	6.22
6	30	75	2.5	6.70	3.00	6.77	6.14	6.71	6.28
7	30	75	2.5	6.66	3.00	6.83	5.65	6.80	7.12

*pH and cell densities (Log cfu/g) were measured at the end of the fermentation*.

For each strain, the pH and cellular concentration reached at the end of the fermentation time are reported according to all experimental conditions and were measured as an exploratory dataset on the microbial response upon adaptation to these conditions.

Considering the strains with an optimum temperature of 37°C, samples fermented with *L. rhamnosus* 1473, showed the highest microbial load in conditions n.5 to n.7, corresponding to the optimal growth temperature of the strains, a medium level of time of fermentation (75 h), and glucose addition. On the other hand, condition n.4, where all factors were set at the maximum values, led to a decrease in microbial concentration from the original inoculum, due to cell lysis. Variations in pH are not easy to interpret, since generally microbial growth is associated with a pH decrease, but the extent of acidification can vary according to growth conditions and substrate. The control sample (seeds and without seeds) has a pH of around 6.5. In conditions n.1, 2, and 3 where the microbial load is similar to the inoculum (7 Log cfu/g), a marked pH decrease was observed only for condition n.2. For this reason, the pH response was difficult to model and predict by the software, so we focused mostly on cell concentration-response.

The Response Surface Methodology (RSM) is a valid tool as it defines the effect of the variables in combination, and it permits the description of the fermentation process for all the strains.

**Figure 3:**
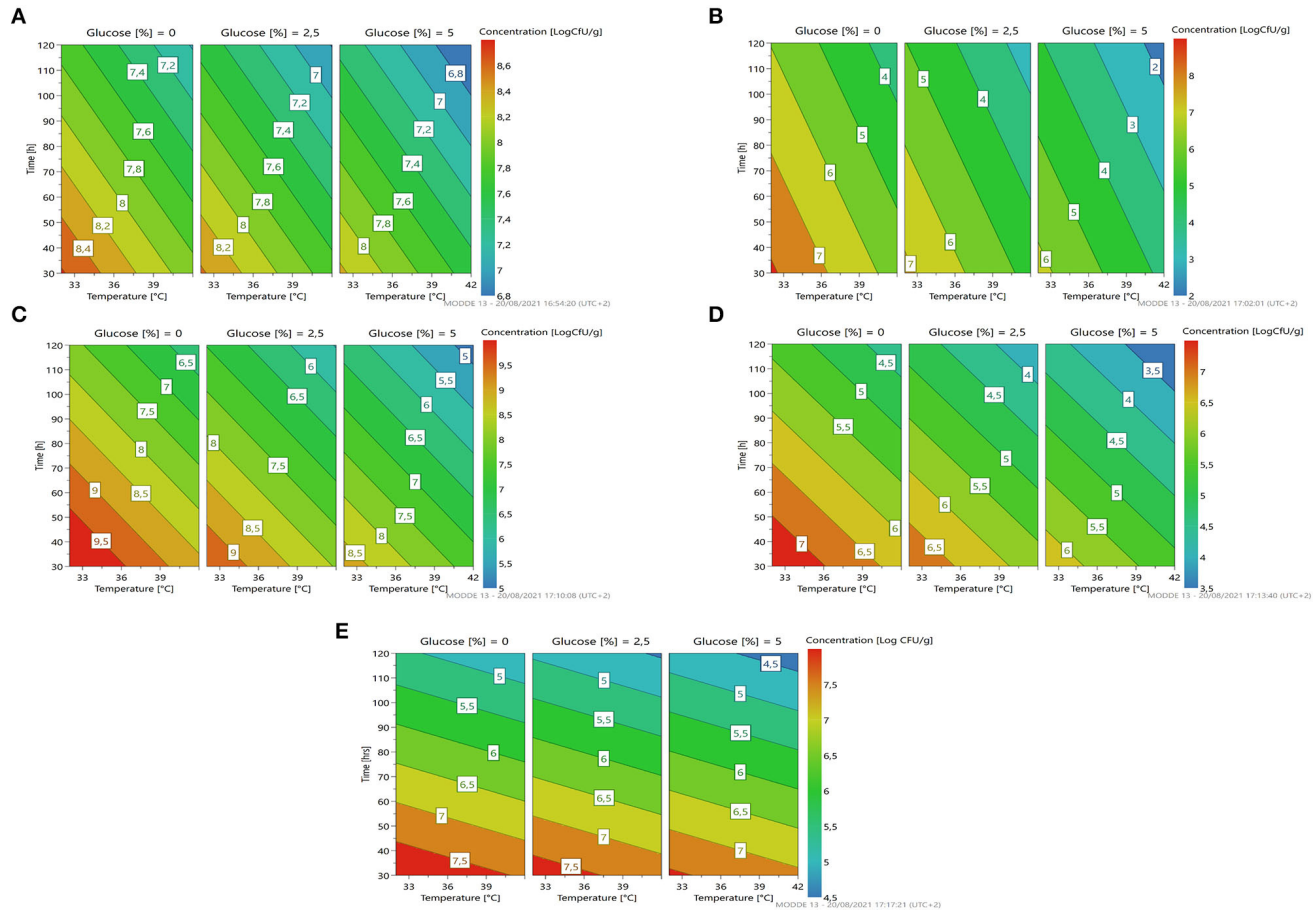
A response contour plot of concentration (Log cfu/g) of *Lacticaseibacillus rhamnosus* 1473 **(A)**; *Lacticaseibacillus paracasei* 4186 **(B)**; *Lacticaseibacillus casei 2240* **(C)**; *Lactiplantibacillus plantarum* 4932 **(D)**; *Pediococcus acidilactici* 3992 **(E)** in samples without seeds, with time and temperature as main factors and with glucose variation.

Looking at Figure 3A, we can see that the percentage of glucose is the factor that most regulates the change in the growth of the microorganism. At a lower glucose concentration, there is a higher growth prediction (red/orange zone). Even temperature and time of fermentation, if reduced, lead to a greater increase in cell concentration of the strain.

This information was collected and used by the software to generate optimization conditions.

Experiments conducted with *L. paracasei* 4186, highlight different trends (Table 1). In this case, the strain shows lower adaptability to the substrates, and only in condition n.1 an increase in microbial load was observed, while in most of the other cases there was cell lysis. Also, for this microorganism, the value of pH decreased in condition n.2, even if no growth was recorded.

As the response surface model shows (Figure 3B), the maximum growth is possible by setting the minimum time and temperature of fermentation without the addition of glucose.

Fermentation with *L. casei* 2240 highlights a similar trend as for *L. rhamnosus* 1473. In this case, the strain was able to grow and replicate in almost all conditions except for the n.3 and n.4, with a long time of fermentation, where cell lysis occurs. Differently from the previous cases, here there is a different trend for pH value. In all conditions, it was recorded a lower pH value even in condition n.4. Only in the first experiment (n.1) did the pH remain unchanged even if an increase in microbial load is observed. As reported in Figure 3C, we noticed as also for *L. casei* 2240 the maximum response of microbial concentration is predicted with minimum addition of glucose and less time of fermentation (red zone).

Experiments conducted with *L. plantarum* 4932 show no significant growth for all conditions. The three center points (n. 5, 6, and 7) highlight a great reproducibility of the model but also indicates that the optimal condition was not sufficient for the strain's growth. In this case, the value of pH decreased under the fermentation conditions n.1, n.2, and n.3. The response surface (Figure 3D) gives us a similar indication as to the previous, reducing the concentration of glucose, time, and temperature of fermentation gives an increase in microbial load, even if is not the most suitable species for *L. paracasei*.

The last bacteria with an optimum temperature of 37°C was *P. acidilactici* 3992 (Figure 3E). This microorganism showed a similar trend to the previous one, with cellular lysis for almost all conditions and acidification of the substrates, especially in condition n. 2.

Moving to the strains with the optimum at 30°C we noticed a different trend, compared to the previous ones (optimum 37°C), in the measured pH values. For these strains, no decrease in pH value was recorded in any of the tested conditions (Table 2).

In general, no growth was observed for all the strains. Moreover, condition n.4 seems where all the factors were set to the higher levels, seems to be the more stressful for these microorganisms, indeed the microbial load decreases, reaching values of about 3–4 Log cfu/g.

**Figure 4:**
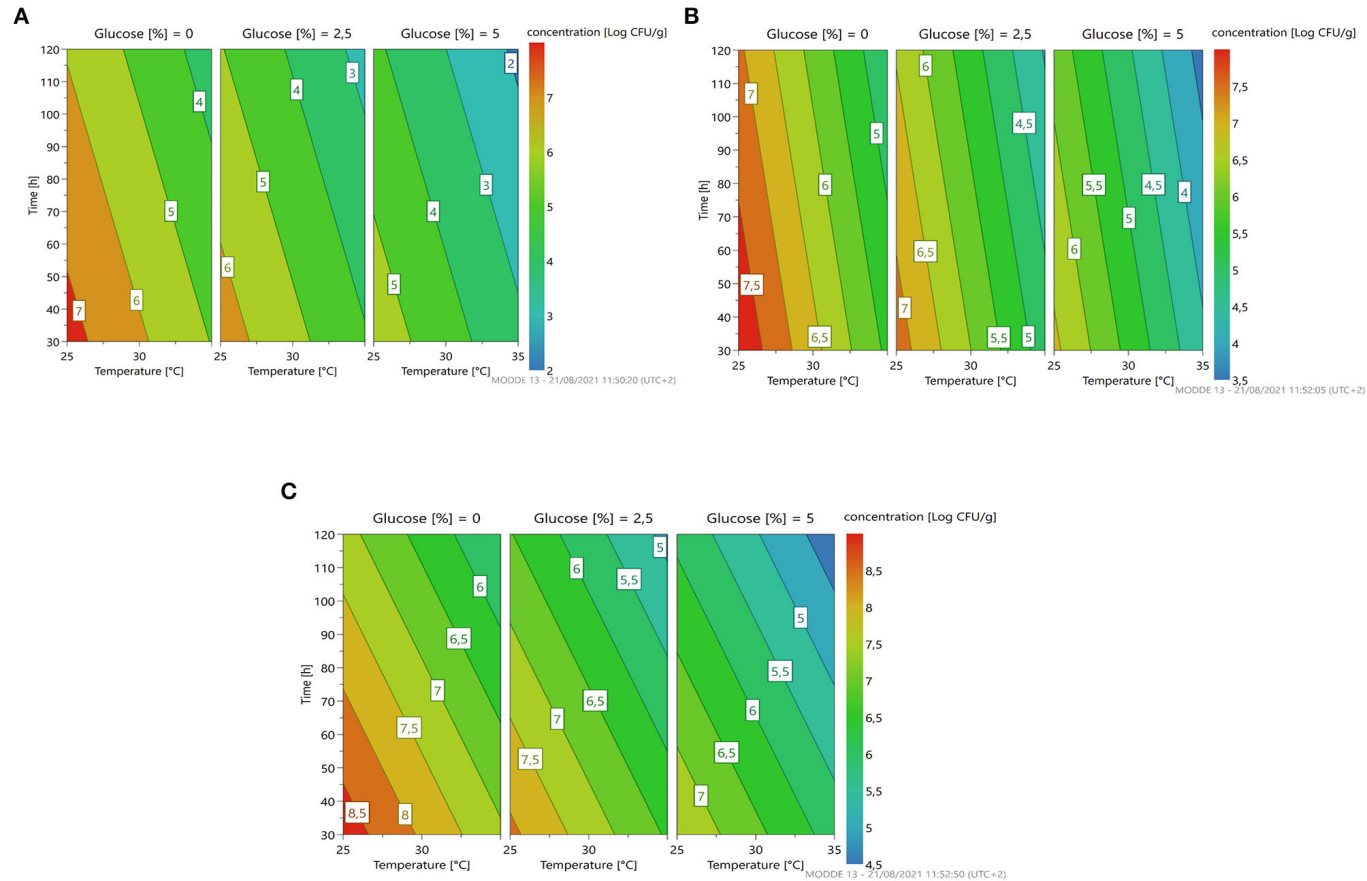
A response contour plot of concentration (Log cfu/g) of *Leuconostoc citreum* 4452 **(A)**; *Leuconostoc mesenteroides* 2194 **(B)**; *Weissella minor* 4451 **(C)** in samples without seeds, with time and temperature as main factors and with glucose variation.

The response surface (Figure 4) gives us an indication for optimization similar to the previously described microorganism: less time, temperature, and concentration of glucose predicting a microbial growth of about 7–7.5 for *L. citreum* 4452 (4A) and *L. mesenteroides* 2194 (4B), while for *W. minor* 4451 it is possible to reach a higher microbial load of about 8.5 Log cfu/g.

At the end of the experiments for the single strain cultures, we exploited the software's optimization function to predict the best growing conditions to use with co-cultures. To reach the highest microbial load (8 Log cfu/g), the software predicted the experimental conditions in which to cultivate the single strains, to perform a validation step. After the validation of these conditions, the co-cultures were made by combining two strains at a time, evaluating all possible binary combinations of strains with the same optimal growth temperature. For the *Lacticaseibacillus* genus, only the *L. casei* 2240 strain was considered, and not *L. paracasei*, because the first had shown greater adaptability to the substrate. All the studied co-cultures are summarized in Table 3.

**Table 3:** Experimental conditions of fermentations with co-cultures in samples without seeds.

**Co-culture**	**Tem. (°C)**	**Time (h)**	**Glucose (%)**	**pH**	**Log (cfu/g)**
*Lacticaseibacillus rhamnosus* 1473 + *Lacticaseibacillus casei* 2240	32	30	0.03	7.01	8.28
*Lacticaseibacillus rhamnosus* 1473 + *Lactiplantibacillus plantarum* 4932	32	30	0.45	7.03	8.21
*Lacticaseibacillus rhamnosus* 1473 + *Pediococcus acidilactici* 3992	32	30	0.03	7.08	8.18
*Lacticaseibacillus casei* 2240 + *Lactiplantibacillus plantarum* 4932	32	30	0.06	7.03	8.18
*Lacticaseibacillus casei* 2240 + *Pediococcus acidilactici* 3992	32	30	0	7.08	7.88
*Lactiplantibacillus plantarum* 4932 + *Pediococcus acidilactici* 3992	32	30	0.06	6.86	7.92
*Weissella minor* 4451 + *Leuconostoc mesenteroides* 2294	25	30	0.06	6.96	8.13
*Weissella minor* 4451 + *Leuconostoc citreum* 4452	25	30	0.07	6.93	8.06
*Leuconostoc citreum* 4452 + *Leuconostoc mesenteroides* 2294	25	30	0.08	6.98	7.65

*pH and cell densities (Log cfu/g) were measured at the end of the fermentation*.

Interestingly, in all the fermentation with co-cultures, it is recorded an increase in microbial load higher than that observed in monocultures. The reason could be the synergistic effect of the microorganisms, and the choice of efficient fermentation conditions according to experimental modeling. In this set of experiments, no variation in pH was observed.

### Fermentation of Exhausted Vanilla Beans With Seeds (S)

The seven experimental conditions for each strain combining time, the temperature of fermentation, and addition of glucose were performed also for the sample with seeds (Tables 4, 5) for a total of 56 experiments. This by-product, differently from the NS one, contain seeds that can be further separated, but fermentation could represent a strategy for its whole valorization. For this reason, we performed a screening approach on this substrate. Also, in this case, experimental conditions n. 5, 6, and 7 are biological replicates to account for natural variance and represent an intermediate condition for all experimental factors.

**Table 4:** Experimental condition for strains with an optimum at 37°C in vanilla beans with seeds.

				***Lacticaseibacillus*** ***rhamnosus*** **1473**		***Lacticaseibacillus paracasei*** **4186**		***Lacticaseibacillus casei*** **2240**		***Lactiplantibacillus plantarum*** **4932**		***Pediococcus acidilactici*** **3992**	
**Exp n**.	**Tem. (°C)**	**Time (h)**	**Glucose (%)**	**pH**	**Log (cfu/g)**	**pH**	**Log (cfu/g)**	**pH**	**Log (cfu/g)**	**pH**	**Log cfu/g**	**pH**	**Log (cfu/g)**
1	32	30	5	6.89	7.27	7.02	7.15	6.52	8.32	6.66	7.32	5.26	7.18
2	42	30	0	6.92	7.25	7.10	6.88	6.66	8.18	6.71	7.18	4.88	5.57
3	32	120	0	6.83	7.62	6.94	5.69	6.73	7.40	6.54	4.83	5.01	6.46
4	42	120	5	6.70	6.04	6.97	4.00	5.31	7.39	6.60	4.00	6.10	4.00
5	37	75	2.5	6.21	8.07	7.18	5.00	6.13	8.01	6.57	6.38	6.46	5.61
6	37	75	2.5	6.11	8.31	7.06	5.70	6.23	8.13	6.66	6.38	6.53	4.98
7	37	75	2.5	6.33	8.32	6.82	5.81	6.48	8.22	6.71	6.61	6.47	5.56

*pH and cell densities (Log cfu/g) were measured at the end of the fermentation*.

**Table 5:** Experimental condition for microorganism with an optimum at 30°C in vanilla beans with seeds.

				***Leuconostoc citreum*** **4452**		***Leuconostoc mesenteroides*** **2194**		***Weissella minor 4451***	
**Exp n**.	**Tem. (°C)**	**Time (h)**	**Glucose (%)**	**pH**	**Log (cfu/g)**	**pH**	**Log (cfu/g)**	**pH**	**Log (cfu/g)**
1	25	30	5	6.96	7.10	6.85	7.33	7.01	7.27
2	35	30	0	6.82	6.20	6.93	5.16	6.89	4.94
3	25	120	0	6.88	7.41	6.93	6.08	6.72	7.17
4	35	120	5	6.95	4.00	6.95	4.00	6.62	4.00
5	30	75	2.5	6.94	6.92	6.97	5.88	6.75	6.73
6	30	75	2.5	7.00	6.84	7.08	5.88	7.06	6.73
7	30	75	2.5	7.01	6.61	7.15	6.18	7.08	6.90

*pH and cell densities (Log cfu/g) were measured at the end of the fermentation*.

Adaptation of the bacterial strains to these substrates was similar to that observed in samples without seeds. In particular, *L. rhamnosus* 1473 and *L. casei* 2240 are the best starters because they can grow, increasing the concentration of 1 Log cfu/g in almost all conditions for *L. casei* 2240, and in the optimal ones for *L. rhamnosus* 1473. Overall, regardless of growth, there is no lowering of pH except in condition n.2 during the fermentation with *P. acidilactici* 3992. The extreme condition (n.4) also in this case negatively affects the growth of *L. paracasei, L. plantarum*, and *P. acidilactici* as for the fermentation in vanilla beans without seeds.

**Figure 5:**
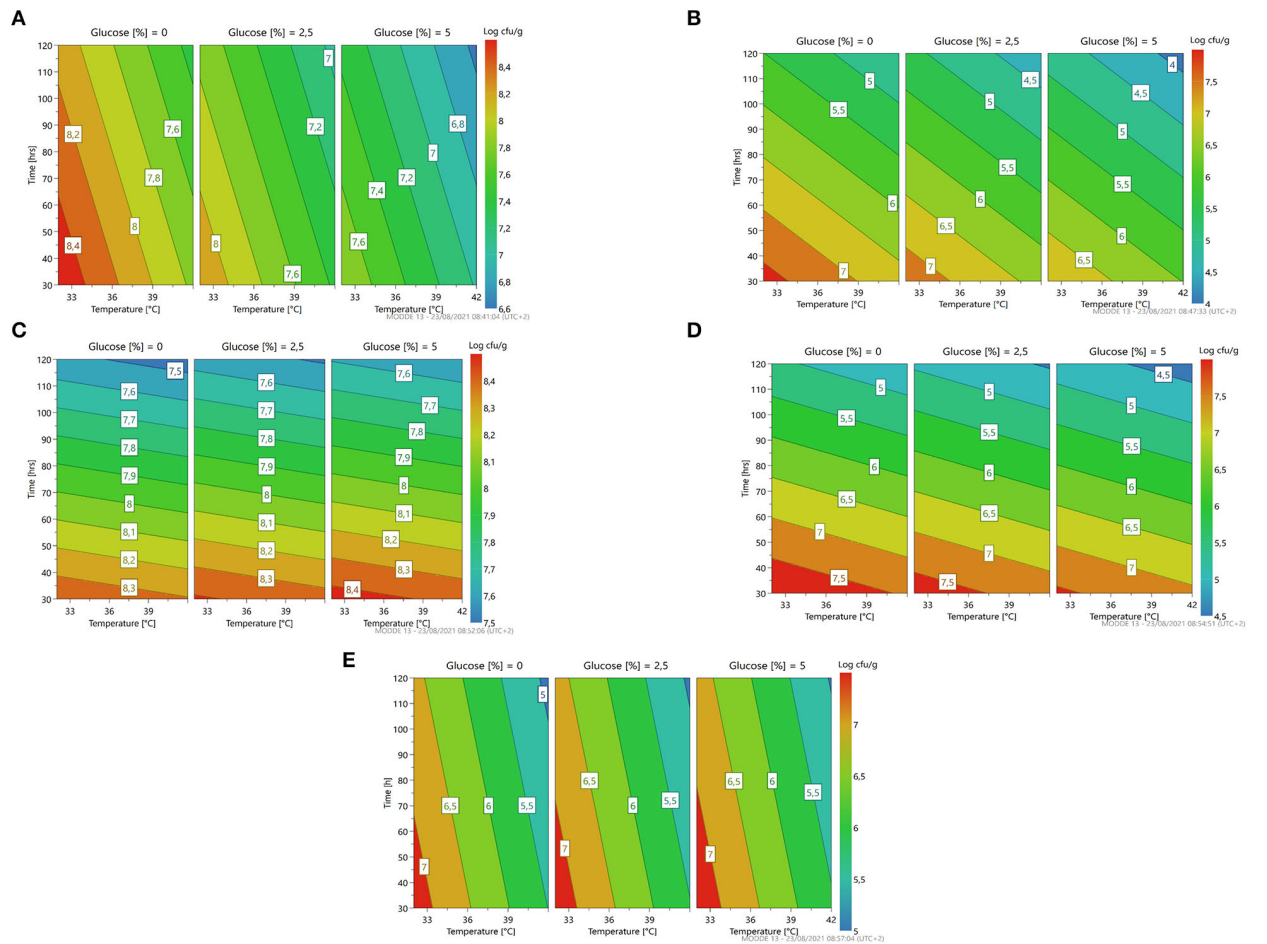
A response contour plot of concentration (Log cfu/g) of *Lacticaseibacillus rhamnosus* 1473 **(A)**; *Lacticaseibacillus paracasei* 4186 **(B)**; *Lacticaseibacillus casei* 2240 **(C)**; *Lactiplantibacillus plantarum* 4932 **(D)**; *Pediococcus acidilactici* 3992 **(E)** in samples with seeds, with time and temperature as main factors and with glucose variation.

The response surface (Figure 5) highlighted the same trend with respect to the fermentation in vanilla beans without seeds. In general, all the parameters could be set at minimum levels to lead to a higher microbial load.

Regarding the strains with an optimum growth temperature of 30°C (Table 5), the best condition seems to be the one with a temperature of 25°C. For *L. citreum* and *W. minor*, the concentration of sugar and fermentation time does not seem to influence growth and there is a slight increase both in condition n.1 with the highest concentration of glucose, and in condition n.3 with the longer time. While for *L. mesenteroides*, the only condition where a slight increase is observed is condition n.1.

**Figure 6:**
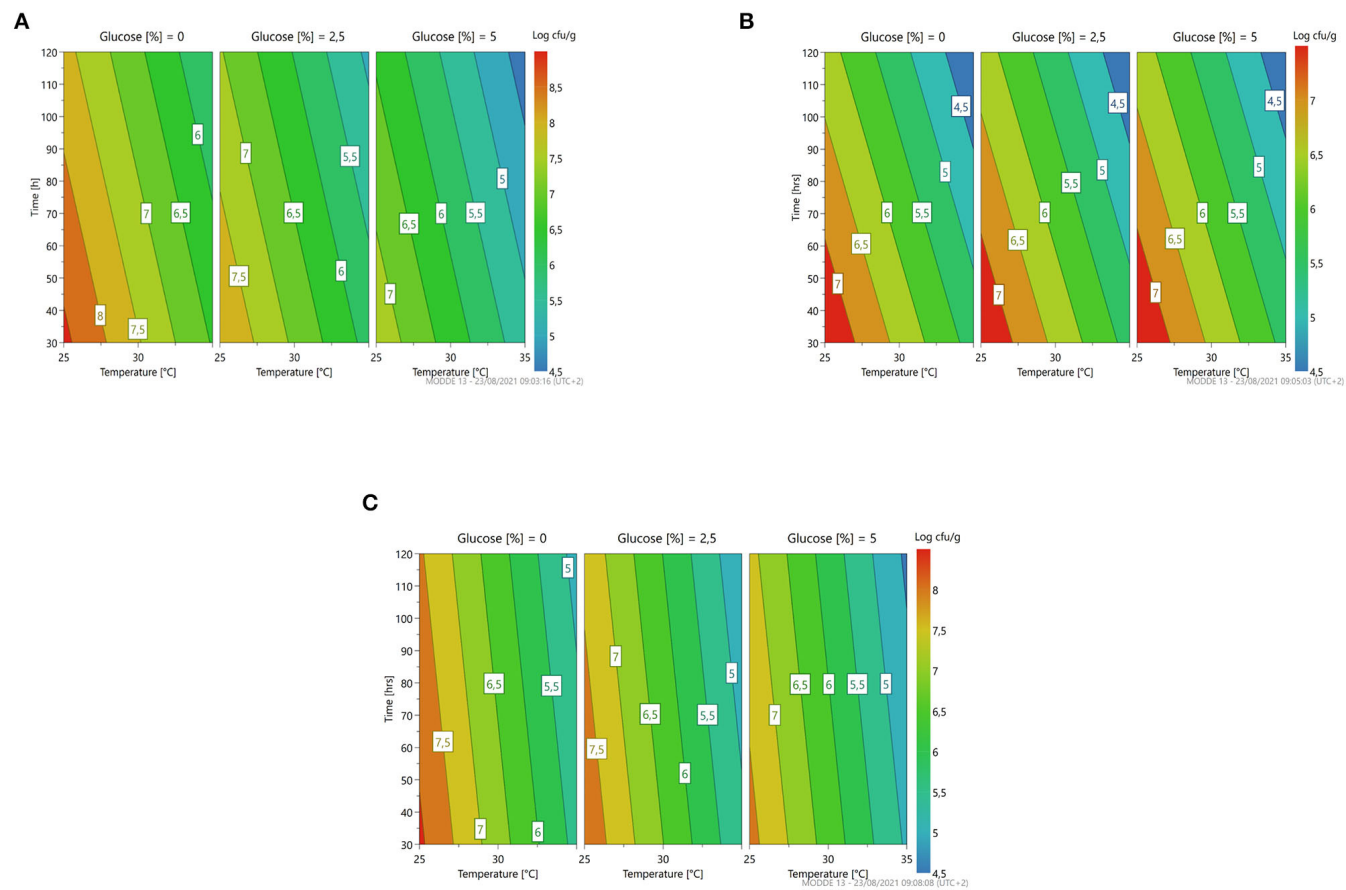
A response contour plot of concentration (Log cfu/g) of *Leuconostoc citreum* 4452 **(A)**; *Leuconostoc mesenteroides* 2194 **(B)**; *Weissella minor* 4451 **(C)** in the sample with seeds, with time and temperature as main factors and with glucose variation.

For all strains, the condition in which all parameters are set at the highest level is the most stressful. Also in this substrate, no variation of pH was recorded during the fermentation with these strains. Figure 6 resumes the response surface generated by the model in which it is possible to observe that *L. mesenteroides* (6B) is the only one that tolerates the addiction of glucose at high concentration, but maintains a bacterial load similar to the inoculum. On the other hand, the response surface suggests that *L. citreum* (6A) and *W. minor* (6C) could reach a higher concentration, about 7.5/8 Log cfu/g (red zone) with no addition of glucose and minimum temperature of fermentation set to 25°C.

As described previously, the single-culture fermentations in vanilla without seeds were followed by co-culture fermentation in the same substrate (Table 6). The software's optimization function predicts the best growing conditions, to use in the validation step. To reach the highest microbial load (8 Log cfu/g), the software gave us the experimental conditions in which to operate. After the validation of these conditions, the co-cultures were made by combining two strains at a time, evaluating all possible combinations, and combining strains with the same optimal growth temperature.

**Table 6:** Condition of fermentation with co-cultures in samples with seeds.

**Co-culture**	**Tem. (°C)**	**Time (h)**	**Glucose (%)**	**pH**	**Log Cfu/g**
*Lacticaseibacillus rhamnosus* 1473 + *Lacticaseibacillus casei* 2240	32	30	0	7.04	8.08
*Lacticaseibacillus rhamnosus* 1473 + *Lactiplantibacillus plantarum* 4932	32	30	0	7.05	8.00
*Lacticaseibacillus rhamnosus* 1473 + *Pediococcus acidilactici* 3992	32	30	0	7.05	7.94
*Lacticaseibacillus casei* 2240 + *Lactiplantibacillus plantarum* 4932	32	30	0	7.10	7.92
*Lacticaseibacillus casei* 2240 + *Pediococcus acidilactici* 3992	32	30	0	7.09	8.02
*Lactiplantibacillus plantarum* 4932 + *Pediococcus acidilactici* 3992	32	30	0	7.12	7.75
*Weissella minor* 4451 + *Leuconostoc mesenteroides* 2294	25	30	0	6.91	7.88
*Weissella minor* 4451 + *Leuconostoc citreum* 4452	25	30	0	6.95	7.86
*Leuconostoc citreum* 4452 + *Leuconostoc mesenteroides* 2294	25	30	0	7.01	7.57

*pH and cell densities (Log cfu/g) were measured at the end of the fermentation*.

The growth is observed for all the co-cultures, also in this case reaching higher values than in fermentation with monoculture. The best co-culture starters combination seems to be the ones composed of *L. rhamnosus* 1473 and *L. casei* 2240; *L. rhamnosus* 1473 and *L. plantarum* 4932; *L. casei* 2240 and *P. acidilactici* 3992.

### Sensory Evaluation

The previously described screening led to the selection of strains combination for sensory evaluation. We selected the combination of strains *L. rhamnosus* 1473 and *L. plantarum* 4932 since these strains had shown a marked difference between the monoculture (Tables 1, 4) and co-culture experiments (Tables 3, 6). We chose conditions n.2 and n.5 which are associated with the extreme and optimal conditions which led to a greater difference in cell concentration. Sensory evaluation was applied to understand if the fermentation can modify the taste of the substrates, opening the way for food applications.

**Figure 7:**
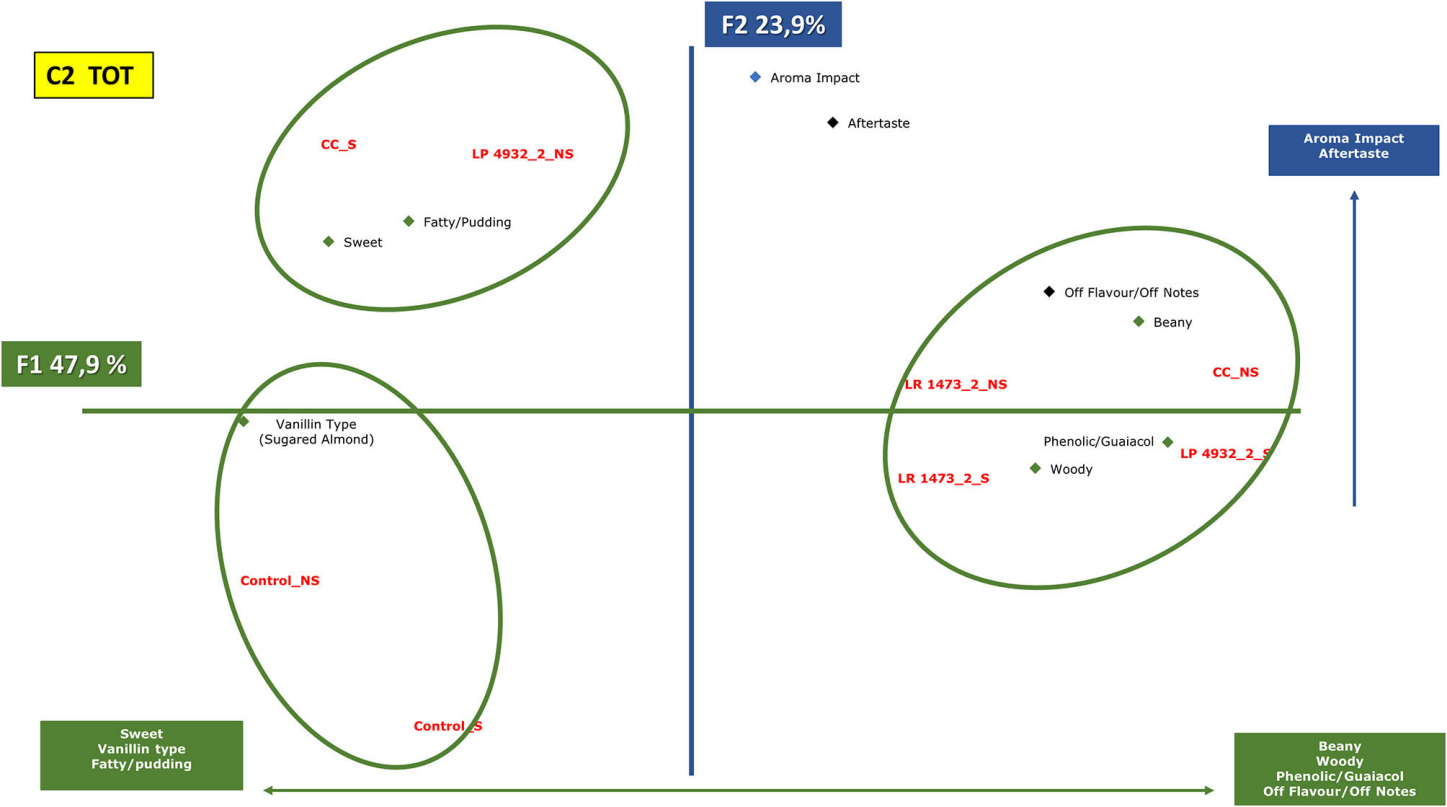
Principal component analysis plot of the similarities and differences in the sensory profiling characteristics of exhausted vanilla beans prepared follow the condition n.2 of DoE and co-culture. CC, co-culture; LP, *Lactiplantibacillus plantarum* 4932; LR, *Lacticaseibacillus rhamnosus* 1473; Control, unfermented samples. S: sample with seeds; NS: sample without seeds.

**Figure 8:**
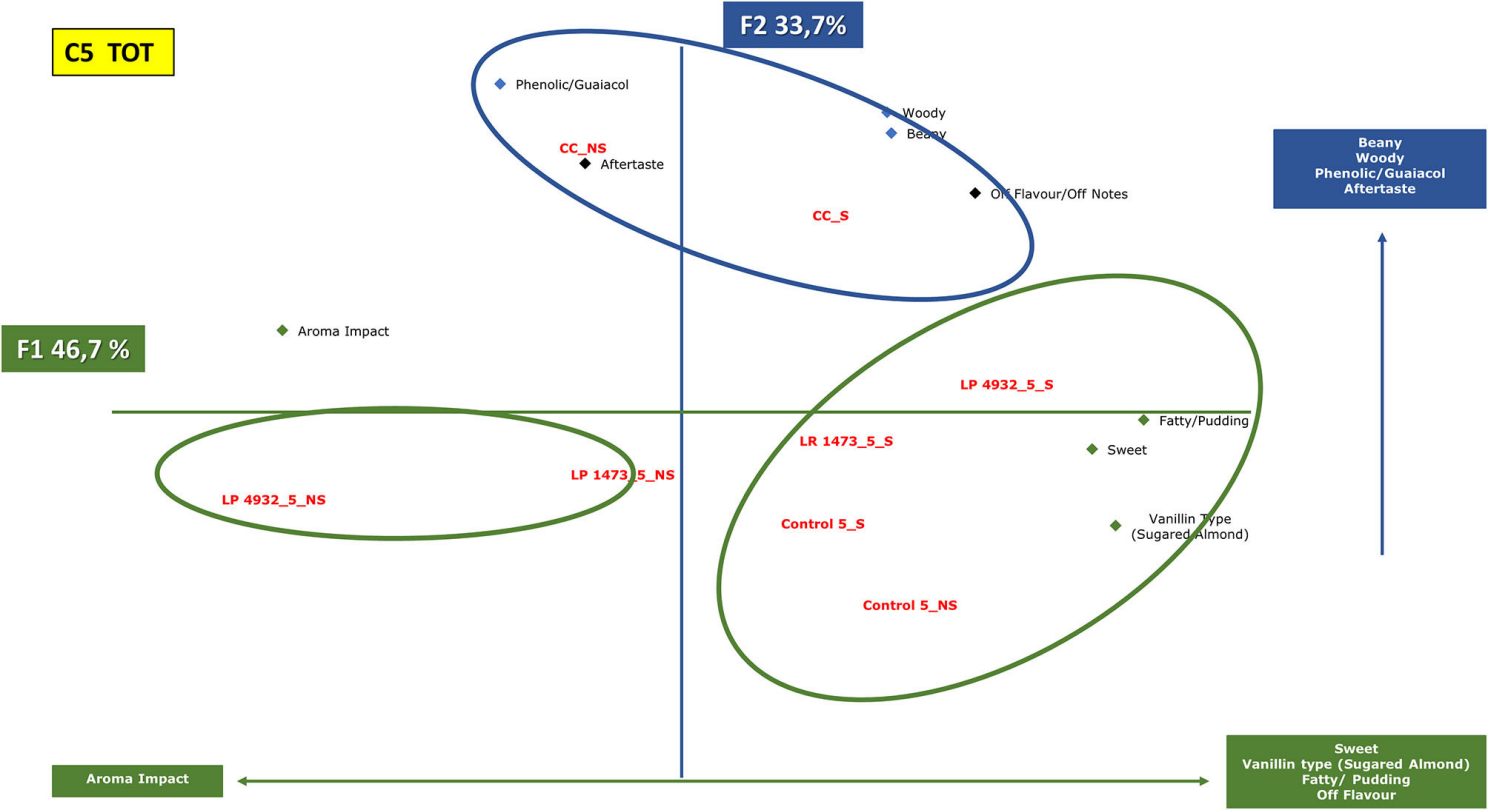
Principal component analysis plot of the similarities and differences in the sensory profiling characteristics of exhausted vanilla beans prepared follow the condition n.5 of DoE and co-cultures.CC, co-culture; LP, *Lactiplantibacillus plantarum* 4932; LR, *Lacticaseibacillus rhamnosus* 1473; Control, unfermented samples. S: sample with seeds; NS: sample without seeds.

In the first tasting (Figure 2), unfermented and fermented samples with *L. rhamnosus* 1473 and *L. plantarum* 4932 and their co-culture were examined by panelists. Panelists were required to smell and taste the products and group them by similarity and/or difference, according to their personal criteria, associating each group with descriptive terms. To obtain more information on the parameters that could influence differences and similarities among the fermented samples in the two conditions (n.2 e n.5), the descriptors identified after tasting were used as variable vectors for Principal Component Analysis (PCA) reported in Figures 7, 8. More details about the QDA analysis of the samples in conditions n.2 and n.5 are presented in Supplementary Figures 1, 2.

Figure 7 represents the samples fermented with condition n.2 together with co-cultures. The first component (47.9% of the information) is mainly characterized by descriptors such as sweet, vanillin type, fatty, and pudding on one side, and beany, woody, phenolic, guaiacol, and off-flavors on the other side. The second component (23.9% of the information) is characterized by a higher aroma impact and aftertaste on the upper side.

From the analysis, three groups were highlighted. The first group is the one composed of control samples, with and without seeds, these were judged by panelists as sweet and vanillin taste but with less aroma impact and aftertaste.

The second group is represented by co-culture with seeds and sample fermented with *L. plantarum* 4932 without seeds. These samples, like the previous ones, are described with vanillin and sweet notes, but in this case with a higher aroma impact.

The third group comprehends samples fermented with *L. rhamnosus* in both substrates (seeds and without), *L. plantarum* with seeds, and co-culture without seeds. These samples are described with beany, woody, phenolic, and guaiacol notes.

Figure 8 shows samples fermented under condition n.5 together with co-cultures. The first axis (46.7% of the information) is mainly characterized by the aroma impact of the samples on one side, and descriptors such as sweet, vanillin type, fatty, and pudding on the other side. The second component (33.7% of the information) is characterized by beany, woody, phenolic, guaiacol, and aftertaste.

In this condition, samples form three distinctive clusters as in the previous, but a different trend is highlighted.

Substrates without seeds, fermented with monoculture, are in the same area of the graph characterized by aroma impact. On the other side, substrates with seeds fermented with the same strains lay on the opposite side of the figure, characterized by sweet, vanillin, pudding notes.

Co-cultures cluster separately in another region of the graph and are characterized by beany, woody, and guaiacol notes.

### Volatile Profile

**Figure 9:**
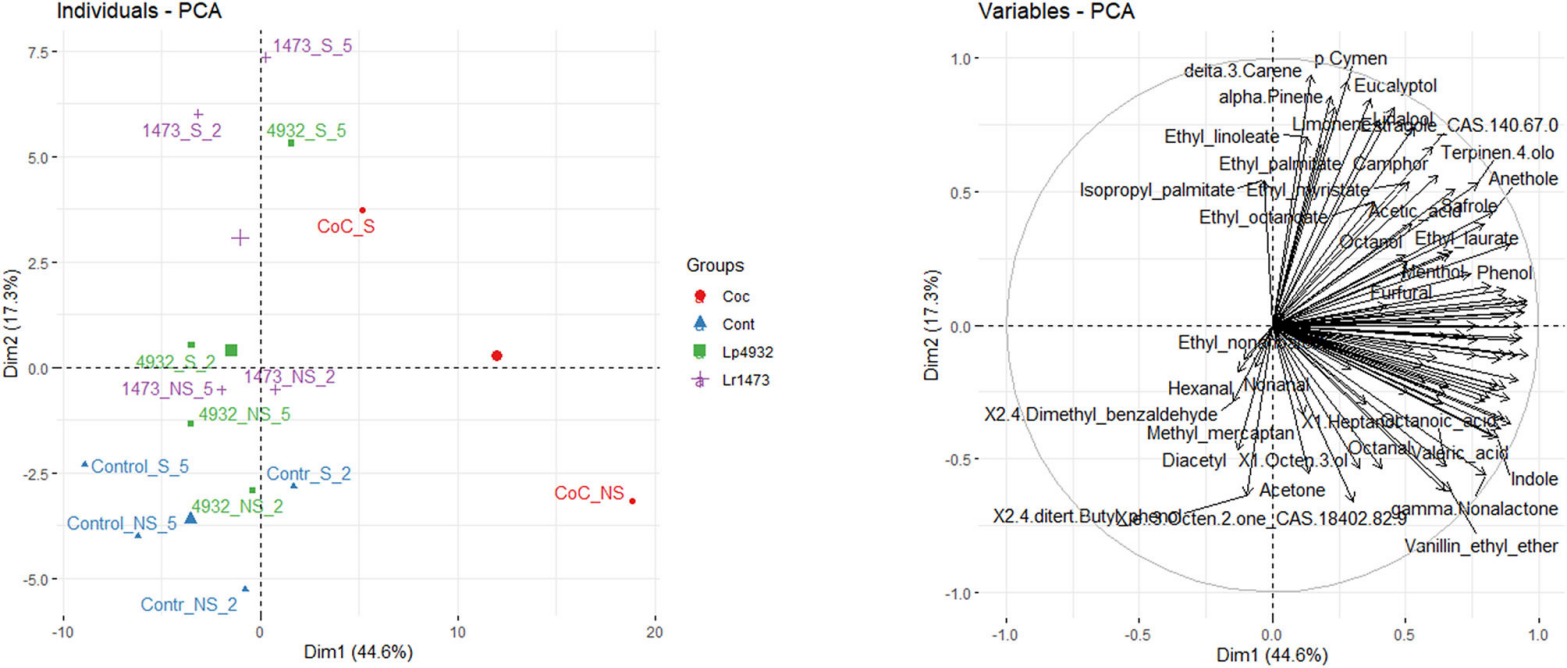
Principal component analysis plot of volatile compounds measured in fermented and unfermented samples of exhausted vanilla beans. Names present in the plot are abbreviated as follows. 1473, *Lacticaseibacillus rhamnosus* 1473; 4932, *Lactiplantibacillus plantarum* 4932; CoC, co-culture of *L. rhamnosus* and *L. plantarum*; S, sample with seeds; NS, sample without seeds; 2, sample fermented using condition n.2 of DoE; 5, sample fermented using condition n.5 of DoE; Control, unfermented sample.

From the analysis of volatile fractions on fermented samples with *L. rhamnosu*s 1473 and *L. plantarum* 4932 in mono and co-culture, and unfermented samples for both conditions (n.2 and n.5), a total of 87 volatile compounds have been identified and quantified using SPME-GC/MS technique, as reported in Supplementary Table 1. A PCA analysis (Figure 9) was performed on these data, and the first two components were plotted, describing 44.6% and 17.3% of the total variability, respectively. As shown in Figure 9, the samples corresponding to S substrate fermented by single and co-culture tends to cluster on top of the PCA, and the separation is driven mostly by ester compounds and terpenes. On the lower left quadrant of the PCA graph, NS samples cluster with all the control samples, while the NS samples fermented with monocultures form a small cluster at the center of the plot, and their clustering is driven by compounds belonging to the class of aldehydes. Finally, the NS sample fermented with the coculture lies in the bottom right quadrant, with a volatile profile characterized mostly by organic acids, and by a higher content of vanillin.

Due to the complex volatile profile of the fermented samples, they focused on those that most characterize the aromatic profile of vanilla following the descriptors relating to the sensory evaluation carried out.

As shown in Supplementary Figure 3, the concentration of guaiacol in the fermented S samples appears to be slightly higher than in the control, in condition n.5. In condition n.2, the concentration of guaiacol in fermented samples decreases in comparison to the control both for S and NS substrates. A different trend is recorded for samples fermented with co-cultures where the concentration of all the six compounds increases, in particular in NS substrates. Fermentation with the co-culture of the NS vanilla beans leads to a significant increase of all the volatile compounds, and this explains the neat separation of this sample also in the PCA graph (Figure 9)

Vanillin content increased in the S and NS substrates after the fermentation by *L. rhamnosus*, in condition n.5, and by co-culture.

2-methoxy-4-methyl phenol is higher in the sample fermented by the co-cultures (.06-.07 μg/g) with respect to the others (0.02–0.04 μg/g). The concentration of benzaldehyde remains constant in almost all conditions except for NS samples fermented with *L. rhamnosus* 1473 in condition n.2 and for the co-cultures. In this study, we observed an increase of hexanol after fermentation with *L. plantarum* and co-culture in NS vanilla beans, while in S samples also after fermentation with *L. rhamnosus*, especially in condition n. 5.

The concentration of hexanoic acid deeply decreased after fermentation with *L. rhamnosus* in S substrate in condition n.2. On the other hand, an increase in this organic acid was recorded in the NS sample, especially in the co-culture.

## Discussion

The aim of this study was to verify the fermentability of the exhausted vanilla beans to add value to the by-product, allowing the recovery of high-value molecules, and to evaluate the differences between samples with and without seeds for their possible application.

Statistical modeling using DoE has been a preferred approach when it comes to studying multiple factors. The DoE and the Response Surface Methodology (RSM), in particular, have been of great industrial benefit in simplifying multifactorial experimental design. Indeed, we explored multiple conditions in order to study the different behavior of LAB strains and find the right starters. Moreover, to better understand the modifications made by microorganisms we conducted a preliminary investigation on sensory profiles, to evaluate the possible use in the food company. We applied DoE to understand the best fermentation condition in which to operate, leading to the selection of conditions that are far from the optimal cultivation conditions for the microorganisms. The current experimental design approach points toward setting the parameters at the minimum levels for all the selected conditions: fermentation operated for fewer hours, at temperatures below the optimal and without glucose addition, aiming to advantageous operating conditions for industrial fermentation.

A different behavior among LAB strains was observed as a result of different adaptation to the vanilla substrate. Notably, no significant differences were observed when bacteria were inoculated in the samples with and without seeds, except for the strains *P. acidilactici* 3992, *L. plantarum* 4932, *L. paracasei* 4186, and *L. citreum* 4452. Indeed, cultivation in the substrate without seeds led to a decrease in microbial concentration, which is more evident in the conditions n.4, 5, 6, and 7, all characterized by sugar addition and an incubation time from 75 to 120 h.

On the other hand, the strains *L. rhamnosus* 1473 and *L. casei* 2240 showed good adaptability to both the substrates, increasing the microbial load of about 1 Log cfu/g in almost all conditions. The similarities in the growth phenotype may be linked to the genotypic relatedness of the two species (28). Moreover, the ability of *Lacticaseibacillus* isolates to ferment different vegetable substrates was recently reported (17, 21, 23), highlighting the metabolic variability of strains, capable to modify the flavor, phenolic content, and nutritional profile of the substrates.

Also, there is a strain-specific variability of the growth phenotype in the same substrate, which showed how LAB of the same species can modify the substrate not only as a result of cell growth but also with a not growing phenotype (24).

Interestingly, the fermentation condition n.2 of DoE often leads to a decrease in pH, but only in strains with optimum growth temperature at 37°C. Generally, the decrease of pH is associated with an increase in microbial load, but in this case, it was observed even when cell concentration does not vary or decreases. This could be explained as a mechanism in response to environmental stress, since the temperature was set at the maximum level, despite LAB being reportedly tolerant to higher temperature stress (29). However, Bancalari et al. (30) has shown how exposure of mesophilic *Lactococcus lactis* to temperatures different from the optimal ones not only affected the growth rate but also had an impact on the acidifying capability of the strains. Another reason can be due to the fermentation metabolism performed by heterofermentative *Lacticaseibacillus* strains used in this study, with respect to the switch from dairy to the plant-based substrate, which might lead to variable pH response in some of the selected conditions. This behavior was reported also for the species *L. plantarum* grown in fruit and vegetable juices and showed a great influence of the isolation source and specific metabolic traits of the strains (25).

LAB co-cultures are not widely studied even if they seem advantageous with respect to the mono-cultures due to the synergistic action of the metabolic pathways of the strains involved (31). Strain formulations for co-culture experiments were selected according to the predictive function of the DoE model, combining the strains that are expected to work best under the same operating conditions.

In this study, we demonstrated that the selected strains show increased growth in exhausted vanilla beans, of about 1 Log cfu/g in comparison to the single cultures. This is in agreement with a previous study where the two types of cultivation were compared, highlighting an increased effect of the co-culture on the volatile profile, with the development of distinctive notes with respect to the mono-cultures (19).

Differences between S and NS by-products had no effect on microbial growth, but rather on the aromatic notes and volatile profile. Moreover, during the sensory panel test, the panelists highlighted a different profile for the two substrates: samples without seeds are described as less tasty, with notes of beany, woody, and phenolic, while samples with seeds were evaluated as sweet with vanillin, fatty, pudding notes. In light of the volatile profile of the samples, it can be suggested that the S and NS fermented beans can have different applications, either as ingredients in the food industry or as a starting material for further aromatic compounds extraction.

During the sensory panel test, differences between the fermented samples could be referred to as the fermentation conditions and strains used. Notably, also strains that did not show a significant increase in microbial concentration, like *L. plantarum*, showed the ability to modify the sensory perception of the fermented sample. Variations in the volatile profile in the absence of growth in inoculated substrates were already observed (24), and highlight the importance of a combined approach, including sensory profile evaluation, for the evaluation of strains to be applied in industrial fermentation.

The volatile profile of the substrates, with or without seeds, were affected by fermentation differently, according to the selected conditions. Quantitative analysis showed that the effect of fermentation on the concentration of the principal volatile compounds was not greatly influenced, except for the co-cultures, which showed an increase in the concentration of hexanoic acid, hexanol, and guaiacol. Accumulation of these compounds could be due to an interaction of the metabolic pathways of the strains, as confirmed by the higher microbial load observed in these samples at the end of fermentation. These differences resulted in a distinctive sensory perception that drove the clustering of samples in the PCA analysis of both sensory and volatile data.

## Conclusion

This study explored fermentation as a yet unapplied strategy for the recovery of exhausted vanilla beans, to evaluate its potential to reuse the fermented product in food applications. LAB was screened as potential starters, for their safety, and for their reported capability to positively affect the sensory profile of various plant-based by-products.

The results presented in this work underline the effectiveness of the DoE approach for the screening of combinations of strains and conditions used in the fermentation of vanilla by-products. The integration with the sensory and instrumental analysis of the samples allowed us to have a broader view of the effect of fermentation on the recovery of volatile compounds that are not primarily extracted.

Furthermore, there is an indication that the use of combinations of strains in co-culture for fermentation can lead to the development of distinctive notes in the final product. Investigation of the metabolic network occurring during the co-cultivation of the strains would be required to expand the knowledge of microbial interactions. In agreement with the obtained results, solid-state fermentation can be considered a promising approach for the recovery and valorization of vanilla by-products. Further studies will be oriented toward selecting the appropriate applications for these fermented by-products as ingredients or flavoring agents in food products.

## Data Availability Statement

The raw data supporting the conclusions of this article will be made available by the authors, without undue reservation.

## Author Contributions

CL and AF: conceptualization and writing-original draft. JH and AL: investigation, data curation formal analysis and writing-original draft. FT: investigation, data curation and formal analysis. VB, GG, and EN: writing-review and editing. All authors contributed to the article and approved the submitted version.

## Conflict of Interest

AF and FT are employed by Giotti-McCormick Company. The remaining authors declare that the research was conducted in the absence of any commercial or financial relationships that could be construed as a potential conflict of interest.

## Publisher's Note

All claims expressed in this article are solely those of the authors and do not necessarily represent those of their affiliated organizations, or those of the publisher, the editors and the reviewers. Any product that may be evaluated in this article, or claim that may be made by its manufacturer, is not guaranteed or endorsed by the publisher.
