# Supplementary material for: Exploring the Potential of Lactic Acid Fermentation for the Recovery of Exhausted Vanilla Beans
Source: Front Nutr. 2022 May 19;9:858716. doi: 10.3389/fnut.2022.858716 (PMC9161551; doi:10.3389/fnut.2022.858716)

**Supplementary Figure 2** Sensory profiles of exhausted vanilla beans fermented in condition n.5. CC: co-culture; LP: *L. plantarum* 4932; LR: *L. rhamnosus* 1473; Control: unfermented samples. S: sample with seeds; NS: sample without seeds.


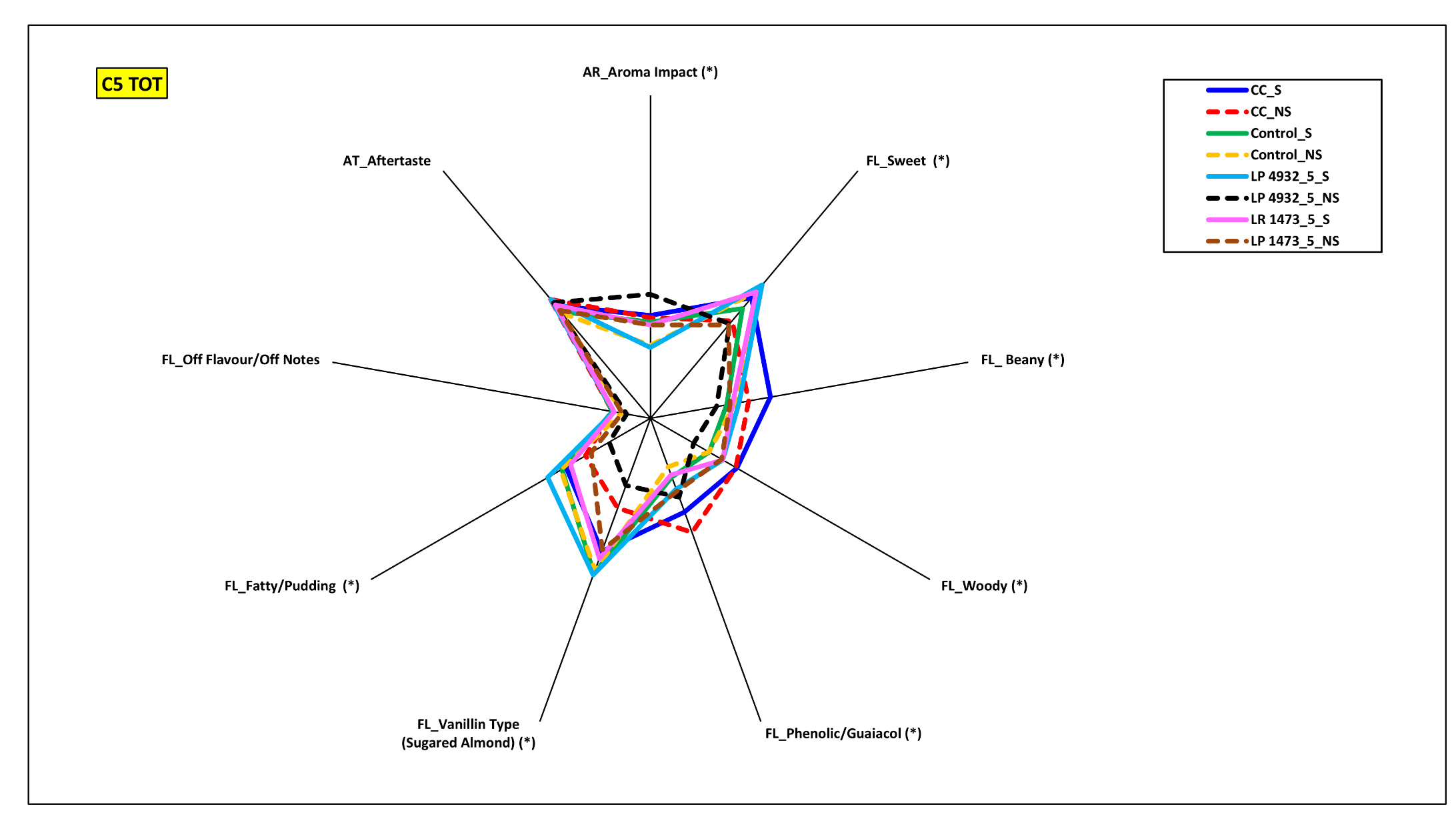

Supplement: Supplementary file 2 [file Table_2.DOCX]
