# Supplementary material for: Exploring the Potential of Lactic Acid Fermentation for the Recovery of Exhausted Vanilla Beans
Source: Front Nutr. 2022 May 19;9:858716. doi: 10.3389/fnut.2022.858716 (PMC9161551; doi:10.3389/fnut.2022.858716)
Supplement: Supplementary file 3 [file Table_3.DOCX]

**Supplementary Table 1** Volatile compounds measured with SPME-GC/MS analysis. Concentration are expressed as ppm.

| Component Name |  | 1473_NS_2 | 4932_NS_5 | 4932_S5 | 4932_NS-2 | Control_NS_5 | Control_NS-2 | CoC_NS | 1473_S_2 | 1473_S_5 | 1473_NS_5 | 4932_S_2 | Control_S_5 | Contr_S_2 | CoC_S |
| --- | --- | --- | --- | --- | --- | --- | --- | --- | --- | --- | --- | --- | --- | --- | --- |
| Methyl_mercaptan |  | 0,00007 | 0,00004 | 0,00003 | 0,00004 | 0,00006 | 0,00005 | 0,00007 | 0,00005 | 0,00005 | 0,00006 | 0,00003 | 0,00007 | 0,00004 | 0,00004 |
| Acetaldehyde |  | 0,00254 | 0,00235 | 0,00172 | 0,00311 | 0,00158 | 0,00336 | 0,00772 | 0,00246 | 0,00305 | 0,00200 | 0,00186 | 0,00135 | 0,00347 | 0,00318 |
| Acetone |  | 0,00050 | 0,00325 | 0,00484 | 0,00285 | 0,00748 | 0,00935 | 0,00531 | 0,00216 | 0,00094 | 0,00410 | 0,00145 | 0,00291 | 0,00986 | 0,00386 |
| Ethyl_acetate |  | 0,00355 | 0,00197 | 0,00289 | 0,00152 | 0,00115 | 0,00195 | 0,00406 | 0,00196 | 0,00359 | 0,00260 | 0,00151 | 0,00192 | 0,00471 | 0,00517 |
| 2-Methyl-2-propanol_CAS:75-65-0 |  | 0,00090 | 0,00064 | 0,00068 | 0,00930 | 0,00098 | 0,00118 | 0,00056 | 0,00184 | 0,00105 | 0,00076 | 0,00569 | 0,00024 | 0,00143 | 0,00089 |
| Diacetyl |  | 0,00037 | 0,00177 | 0,00255 | 0,00300 | 0,00602 | 0,00437 | 0,00302 | 0,00416 | 0,00000 | 0,00321 | 0,00333 | 0,00335 | 0,00681 | 0,00307 |
| alpha-Pinene |  | 0,00064 | 0,00058 | 0,00187 | 0,00070 | 0,00058 | 0,00039 | 0,00095 | 0,00130 | 0,00190 | 0,00123 | 0,00057 | 0,00057 | 0,00062 | 0,00115 |
| Hexanal |  | 0,06024 | 0,06163 | 0,14721 | 0,03555 | 0,10397 | 0,06571 | 0,04066 | 0,01449 | 0,02415 | 0,01681 | 0,02068 | 0,07215 | 0,07135 | 0,02519 |
| delta-3-Carene |  | 0,00171 | 0,00105 | 0,00372 | 0,00111 | 0,00056 | 0,00061 | 0,00127 | 0,00312 | 0,00432 | 0,00149 | 0,00113 | 0,00061 | 0,00101 | 0,00209 |
| Heptanal |  | 0,01651 | 0,01618 | 0,01461 | 0,01169 | 0,00734 | 0,01797 | 0,02574 | 0,00907 | 0,01131 | 0,01104 | 0,00974 | 0,00509 | 0,02031 | 0,01495 |
| Limonene |  | 0,09989 | 0,12495 | 0,23721 | 0,10141 | 0,08600 | 0,05243 | 0,19497 | 0,25177 | 0,34226 | 0,22058 | 0,11038 | 0,09036 | 0,08043 | 0,14554 |
| Eucalyptol |  | 0,00475 | 0,00416 | 0,00845 | 0,00448 | 0,00418 | 0,00413 | 0,00586 | 0,00763 | 0,00845 | 0,00450 | 0,00486 | 0,00362 | 0,00618 | 0,00834 |
| 2-Pentyl_furan |  | 0,01281 | 0,00675 | 0,02745 | 0,00845 | 0,00929 | 0,00749 | 0,02354 | 0,00340 | 0,01332 | 0,01336 | 0,00816 | 0,00855 | 0,01031 | 0,02176 |
| p-Cymen |  | 0,01120 | 0,01068 | 0,02527 | 0,00920 | 0,00494 | 0,00550 | 0,01319 | 0,01823 | 0,02947 | 0,01316 | 0,01045 | 0,00509 | 0,00891 | 0,01853 |
| Octanal |  | 0,02361 | 0,02198 | 0,01412 | 0,02295 | 0,01725 | 0,02210 | 0,02396 | 0,01705 | 0,01279 | 0,01236 | 0,01322 | 0,01122 | 0,02027 | 0,01300 |
| (e)-2-Heptenal |  | 0,04818 | 0,03728 | 0,06063 | 0,03487 | 0,04237 | 0,03792 | 0,05567 | 0,01247 | 0,01689 | 0,02778 | 0,02180 | 0,03600 | 0,04428 | 0,05814 |
| Hexyl_alcohol |  | 0,03052 | 0,07751 | 0,05106 | 0,10165 | 0,01334 | 0,01247 | 0,10668 | 0,01824 | 0,04891 | 0,02620 | 0,04258 | 0,00976 | 0,01707 | 0,05851 |
| Nonanal |  | 0,15561 | 0,07309 | 0,05347 | 0,03495 | 0,05357 | 0,05615 | 0,04455 | 0,06112 | 0,03170 | 0,02638 | 0,05820 | 0,04874 | 0,08338 | 0,03093 |
| (e)-3-Octen-2-one_CAS:18402-82-9 |  | 0,02153 | 0,01372 | 0,01986 | 0,01829 | 0,03594 | 0,04196 | 0,03627 | 0,00241 | 0,00909 | 0,01764 | 0,00778 | 0,02935 | 0,04302 | 0,03204 |
| 5-Ethylcyclopent-1-ene_carboxaldehyde |  | 0,01284 | 0,00755 | 0,00768 | 0,00530 | 0,00628 | 0,00606 | 0,01216 | 0,00214 | 0,00475 | 0,00596 | 0,00374 | 0,00343 | 0,00588 | 0,00670 |
| alpha-Thujone |  | 0,00921 | 0,00827 | 0,01211 | 0,00664 | 0,00545 | 0,00551 | 0,00955 | 0,00982 | 0,01370 | 0,00670 | 0,00646 | 0,00512 | 0,00840 | 0,01221 |
| beta-Thujone |  | 0,01325 | 0,00834 | 0,01458 | 0,00890 | 0,00873 | 0,00826 | 0,01122 | 0,01169 | 0,01730 | 0,00751 | 0,00959 | 0,00701 | 0,01080 | 0,01582 |
| Ethyl_octanoate |  | 0,03083 | 0,02326 | 0,09677 | 0,01945 | 0,01691 | 0,01756 | 0,03932 | 0,01129 | 0,03562 | 0,02047 | 0,02197 | 0,02483 | 0,03075 | 0,06441 |
| 1-Octen-3-ol |  | 0,07216 | 0,07421 | 0,10633 | 0,05665 | 0,11748 | 0,13434 | 0,11897 | 0,02132 | 0,03253 | 0,05576 | 0,03572 | 0,08976 | 0,14737 | 0,11622 |
| 1-Heptanol |  | 0,02941 | 0,02702 | 0,01774 | 0,02729 | 0,01492 | 0,07547 | 0,03509 | 0,02420 | 0,01837 | 0,01766 | 0,01482 | 0,00889 | 0,07610 | 0,05855 |
| Acetic_acid |  | 0,04078 | 0,03379 | 0,06257 | 0,05308 | 0,02431 | 0,03583 | 0,10319 | 0,05496 | 0,10964 | 0,11892 | 0,04939 | 0,02578 | 0,04740 | 0,05558 |
| Furfural |  | 0,07162 | 0,08456 | 0,16185 | 0,06455 | 0,10265 | 0,10474 | 0,19716 | 0,12769 | 0,13340 | 0,19458 | 0,09525 | 0,08715 | 0,09221 | 0,09900 |
| Camphor |  | 0,01426 | 0,01267 | 0,01997 | 0,00983 | 0,01184 | 0,01445 | 0,01818 | 0,01526 | 0,01918 | 0,01012 | 0,01129 | 0,00907 | 0,01623 | 0,02109 |
| 3-Nonen-2-one_CAS:14309-57-0 |  | 0,02466 | 0,01765 | 0,03723 | 0,01507 | 0,01522 | 0,01812 | 0,04299 | 0,00108 | 0,00857 | 0,01724 | 0,01047 | 0,01360 | 0,02116 | 0,04710 |
| Benzaldehyde |  | 0,23200 | 0,15469 | 0,12418 | 0,11105 | 0,12050 | 0,15183 | 0,34531 | 0,12353 | 0,12517 | 0,13992 | 0,11478 | 0,07494 | 0,14093 | 0,13798 |
| (e)-2-Nonenal |  | 0,02222 | 0,01137 | 0,02185 | 0,01353 | 0,01093 | 0,01662 | 0,02320 | 0,00676 | 0,00732 | 0,01031 | 0,01703 | 0,01208 | 0,02079 | 0,02750 |
| Ethyl_nonanoate-IS |  | 0,00000 | 0,00000 | 0,00000 | 0,00000 | 0,00000 | 0,00000 | 0,00000 | 0,00000 | 0,00000 | 0,00000 | 0,00000 | 0,00000 | 0,00000 | 0,00000 |
| Linalool |  | 0,04034 | 0,02798 | 0,03945 | 0,02668 | 0,02760 | 0,02522 | 0,04123 | 0,04099 | 0,04720 | 0,02871 | 0,02936 | 0,02744 | 0,03438 | 0,04664 |
| Octanol |  | 0,21457 | 0,14391 | 0,09031 | 0,11391 | 0,08715 | 0,08663 | 0,22286 | 0,23006 | 0,14167 | 0,11307 | 0,09581 | 0,05565 | 0,09054 | 0,11087 |
| Terpinen-4-olo |  | 0,01879 | 0,01636 | 0,02119 | 0,01553 | 0,01324 | 0,01809 | 0,02464 | 0,02062 | 0,02375 | 0,01580 | 0,01648 | 0,01241 | 0,02062 | 0,02397 |
| (e)-2-Octen-1-ol |  | 0,01651 | 0,01069 | 0,01051 | 0,01101 | 0,01066 | 0,01256 | 0,02203 | 0,01323 | 0,01154 | 0,01080 | 0,00824 | 0,00801 | 0,01282 | 0,01256 |
| Menthol |  | 0,00684 | 0,00564 | 0,00607 | 0,00447 | 0,00648 | 0,00464 | 0,00784 | 0,00561 | 0,00699 | 0,00457 | 0,00478 | 0,00461 | 0,00660 | 0,00732 |
| (e)-2-Decenal |  | 0,07135 | 0,05120 | 0,05190 | 0,05342 | 0,01889 | 0,03716 | 0,11365 | 0,03125 | 0,04039 | 0,04090 | 0,03702 | 0,01746 | 0,05179 | 0,07010 |
| 2,3-Octandione |  | 0,03785 | 0,05493 | 0,06799 | 0,05514 | 0,04144 | 0,04435 | 0,10282 | 0,01230 | 0,02235 | 0,03080 | 0,03710 | 0,02779 | 0,04568 | 0,06351 |
| Nonanol |  | 0,06771 | 0,14579 | 0,09514 | 0,14220 | 0,04441 | 0,04746 | 0,27735 | 0,07271 | 0,07174 | 0,04350 | 0,15190 | 0,03182 | 0,05177 | 0,17197 |
| Estragole_CAS:140-67-0 |  | 0,23498 | 0,16310 | 0,29698 | 0,14275 | 0,09785 | 0,12513 | 0,27071 | 0,23099 | 0,26606 | 0,13628 | 0,20239 | 0,11471 | 0,20908 | 0,38416 |
| Isovaleric_acid |  | 0,01079 | 0,01187 | 0,00957 | 0,01316 | 0,01113 | 0,01312 | 0,02521 | 0,01466 | 0,01235 | 0,01688 | 0,00873 | 0,00745 | 0,01196 | 0,00667 |
| 2-Methyl_butyric_acid |  | 0,00707 | 0,00719 | 0,00591 | 0,00854 | 0,00767 | 0,00918 | 0,01594 | 0,00947 | 0,00757 | 0,01111 | 0,00509 | 0,00493 | 0,00816 | 0,00445 |
| alpha-Terpinyl_acetate |  | 0,00563 | 0,00409 | 0,00533 | 0,00389 | 0,00320 | 0,00368 | 0,00722 | 0,00445 | 0,00492 | 0,00411 | 0,00384 | 0,00258 | 0,00403 | 0,00532 |
| alpha-Terpineol |  | 0,02887 | 0,02442 | 0,02786 | 0,02343 | 0,01732 | 0,02479 | 0,03092 | 0,01765 | 0,02084 | 0,01187 | 0,02195 | 0,01905 | 0,02959 | 0,03130 |
| (e)(e)-2,4-Nonadienal |  | 0,02065 | 0,01799 | 0,02874 | 0,01761 | 0,01589 | 0,02098 | 0,01801 | 0,00963 | 0,01064 | 0,00874 | 0,01499 | 0,01495 | 0,02529 | 0,02203 |
| Carvone |  | 0,01753 | 0,01324 | 0,01617 | 0,01579 | 0,01332 | 0,01523 | 0,02433 | 0,01270 | 0,01519 | 0,01213 | 0,01513 | 0,01169 | 0,01835 | 0,02076 |
| Valeric_acid |  | 0,01602 | 0,01490 | 0,01489 | 0,01767 | 0,01453 | 0,01746 | 0,03386 | 0,00507 | 0,01386 | 0,02091 | 0,01133 | 0,00995 | 0,01661 | 0,01663 |
| 2-Undecenal |  | 0,01568 | 0,00934 | 0,01529 | 0,01436 | 0,01093 | 0,01485 | 0,02400 | 0,00541 | 0,00923 | 0,01173 | 0,01288 | 0,01034 | 0,01612 | 0,02124 |
| Sesquiterpen1 |  | 0,00520 | 0,00409 | 0,00502 | 0,00529 | 0,00311 | 0,00453 | 0,00748 | 0,00415 | 0,00440 | 0,00439 | 0,00450 | 0,00265 | 0,00466 | 0,00590 |
| Methyl_salicylate |  | 0,00530 | 0,00331 | 0,00491 | 0,00332 | 0,00251 | 0,00350 | 0,01293 | 0,00480 | 0,00422 | 0,00295 | 0,00482 | 0,00269 | 0,00541 | 0,01145 |
| 2,4-Dimethyl_benzaldehyde |  | 0,06025 | 0,01824 | 0,01466 | 0,08691 | 0,03279 | 0,04627 | 0,02207 | 0,04664 | 0,01752 | 0,00513 | 0,05862 | 0,02361 | 0,03572 | 0,01403 |
| Anethole |  | 0,04741 | 0,03371 | 0,05012 | 0,03475 | 0,02259 | 0,03075 | 0,06800 | 0,04301 | 0,04666 | 0,02823 | 0,04174 | 0,02217 | 0,04163 | 0,06904 |
| Ethyl_laurate |  | 0,00301 | 0,00325 | 0,00502 | 0,00410 | 0,00177 | 0,00323 | 0,00592 | 0,00440 | 0,00376 | 0,00268 | 0,00393 | 0,00182 | 0,00360 | 0,00446 |
| Hexanoic_acid |  | 0,42250 | 0,37921 | 0,37676 | 0,43045 | 0,34919 | 0,42148 | 0,86436 | 0,11267 | 0,31324 | 0,45265 | 0,24634 | 0,21128 | 0,36707 | 0,42258 |
| Guaiacol |  | 1,03400 | 0,99277 | 1,13330 | 1,13945 | 0,98619 | 1,22768 | 1,87638 | 1,16498 | 1,37911 | 0,96084 | 1,14165 | 0,86776 | 1,52528 | 1,35801 |
| Safrole |  | 0,01740 | 0,01323 | 0,02486 | 0,01251 | 0,00921 | 0,01273 | 0,02419 | 0,01685 | 0,01906 | 0,01174 | 0,01626 | 0,01126 | 0,01967 | 0,03106 |
| Benzyl_alcohol |  | 0,00889 | 0,00716 | 0,00805 | 0,00968 | 0,00667 | 0,00845 | 0,02365 | 0,01154 | 0,01291 | 0,00920 | 0,01093 | 0,00505 | 0,00866 | 0,01075 |
| Phenethyl_alcohol |  | 0,00720 | 0,00616 | 0,00489 | 0,00821 | 0,00474 | 0,00622 | 0,02918 | 0,00694 | 0,01002 | 0,00833 | 0,00653 | 0,00361 | 0,00549 | 0,00759 |
| from_Methyl_cinnamate |  | 0,01203 | 0,01032 | 0,01381 | 0,01272 | 0,00903 | 0,01286 | 0,02382 | 0,01187 | 0,01330 | 0,01080 | 0,01306 | 0,00863 | 0,01482 | 0,01901 |
| 2-Methoxy-4-methyl_phenol |  | 0,03177 | 0,02831 | 0,03675 | 0,03694 | 0,02567 | 0,03664 | 0,07027 | 0,03388 | 0,04337 | 0,03460 | 0,03761 | 0,02376 | 0,04126 | 0,05135 |
| Heptanoic_acid |  | 0,04129 | 0,03732 | 0,03350 | 0,04173 | 0,03397 | 0,04268 | 0,08098 | 0,02703 | 0,03424 | 0,04643 | 0,03059 | 0,02400 | 0,03646 | 0,03714 |
| Phenol |  | 0,01458 | 0,01464 | 0,01939 | 0,01841 | 0,01621 | 0,01948 | 0,03162 | 0,02055 | 0,02462 | 0,01613 | 0,01917 | 0,01498 | 0,02514 | 0,02237 |
| Methyl_eugenol |  | 0,00212 | 0,00179 | 0,00208 | 0,00234 | 0,00172 | 0,00247 | 0,00343 | 0,00189 | 0,00184 | 0,00161 | 0,00234 | 0,00163 | 0,00241 | 0,00255 |
| p-Anisaldehyde |  | 0,00403 | 0,00382 | 0,00499 | 0,00517 | 0,00372 | 0,00569 | 0,00931 | 0,00298 | 0,00345 | 0,00374 | 0,00384 | 0,00317 | 0,00544 | 0,00497 |
| gamma-Nonalactone |  | 0,01116 | 0,01047 | 0,00913 | 0,01346 | 0,01044 | 0,01451 | 0,02232 | 0,00696 | 0,00932 | 0,01068 | 0,01073 | 0,00868 | 0,01331 | 0,01045 |
| Ethyl_myristate |  | 0,00357 | 0,00423 | 0,00534 | 0,00505 | 0,00191 | 0,00402 | 0,00637 | 0,00777 | 0,00514 | 0,00326 | 0,00503 | 0,00173 | 0,00405 | 0,00479 |
| Octanoic_acid |  | 0,05518 | 0,05024 | 0,04119 | 0,04913 | 0,05568 | 0,06394 | 0,10024 | 0,04358 | 0,03584 | 0,07202 | 0,03280 | 0,03598 | 0,05452 | 0,03637 |
| Methyl_cinnamate |  | 0,01222 | 0,01083 | 0,01467 | 0,01457 | 0,00910 | 0,01385 | 0,02365 | 0,01244 | 0,01182 | 0,00962 | 0,01360 | 0,00811 | 0,01496 | 0,01783 |
| 4-Methyl_phenol |  | 0,00751 | 0,00531 | 0,00628 | 0,00736 | 0,00469 | 0,00708 | 0,02392 | 0,00987 | 0,00789 | 0,00696 | 0,00924 | 0,00433 | 0,00738 | 0,01487 |
| Ethyl_cinnamate |  | 0,00311 | 0,00248 | 0,00309 | 0,00344 | 0,00211 | 0,00304 | 0,00550 | 0,00257 | 0,00260 | 0,00240 | 0,00294 | 0,00181 | 0,00310 | 0,00361 |
| Nonanoic_acid |  | 0,18603 | 0,20180 | 0,20240 | 0,19476 | 0,20394 | 0,22672 | 0,46823 | 0,18279 | 0,30253 | 0,34395 | 0,22240 | 0,14729 | 0,29178 | 0,20863 |
| Carvacrol |  | 0,00216 | 0,00195 | 0,00210 | 0,00290 | 0,00200 | 0,00250 | 0,00408 | 0,00226 | 0,00202 | 0,00196 | 0,00193 | 0,00157 | 0,00270 | 0,00247 |
| Isopropyl_palmitate |  | 0,01077 | 0,00528 | 0,02888 | 0,00563 | 0,00431 | 0,00349 | 0,00402 | 0,03119 | 0,00183 | 0,00207 | 0,00246 | 0,00237 | 0,00357 | 0,00385 |
| Ethyl_palmitate |  | 0,00680 | 0,00681 | 0,01222 | 0,00940 | 0,00346 | 0,00751 | 0,01220 | 0,02824 | 0,01484 | 0,00636 | 0,01041 | 0,00401 | 0,00853 | 0,00969 |
| Decanoic_acid |  | 0,01112 | 0,00746 | 0,01227 | 0,00809 | 0,00886 | 0,00971 | 0,01509 | 0,00847 | 0,00869 | 0,01474 | 0,00786 | 0,00478 | 0,01308 | 0,00578 |
| Anisyl_alcohol |  | 0,00166 | 0,00133 | 0,00157 | 0,00177 | 0,00102 | 0,00179 | 0,00478 | 0,00190 | 0,00162 | 0,00191 | 0,00163 | 0,00097 | 0,00173 | 0,00180 |
| 2,4-ditert-Butyl_phenol |  | 0,00356 | 0,01374 | 0,01426 | 0,00632 | 0,04954 | 0,06649 | 0,02447 | 0,00286 | 0,00258 | 0,00977 | 0,00500 | 0,04747 | 0,07190 | 0,01306 |
| Vanillin_ethyl_ether |  | 0,01397 | 0,01369 | 0,00701 | 0,02406 | 0,01202 | 0,01899 | 0,03064 | 0,00653 | 0,00847 | 0,00748 | 0,01047 | 0,00854 | 0,01805 | 0,01133 |
| Coumarin |  | 0,00022 | 0,00026 | 0,00043 | 0,00323 | 0,00023 | 0,00030 | 0,00267 | 0,00021 | 0,00027 | 0,00026 | 0,00217 | 0,00018 | 0,00025 | 0,00267 |
| Benzoic_acid |  | 0,00212 | 0,00186 | 0,00249 | 0,00228 | 0,00219 | 0,00248 | 0,00550 | 0,00237 | 0,00340 | 0,00381 | 0,00184 | 0,00174 | 0,00283 | 0,00197 |
| Indole |  | 0,00080 | 0,00066 | 0,00059 | 0,00111 | 0,00073 | 0,00086 | 0,00176 | 0,00054 | 0,00070 | 0,00067 | 0,00068 | 0,00054 | 0,00087 | 0,00077 |
| Lauric_acid |  | 0,00247 | 0,00149 | 0,00331 | 0,00262 | 0,00141 | 0,00160 | 0,00326 | 0,00190 | 0,00192 | 0,00467 | 0,00259 | 0,00087 | 0,00276 | 0,00186 |
| Ethyl_linoleate |  | 0,00890 | 0,00808 | 0,01393 | 0,01137 | 0,00513 | 0,00849 | 0,01516 | 0,03734 | 0,02283 | 0,00904 | 0,01376 | 0,00595 | 0,01067 | 0,01174 |
| 4-Ethoxymethyl_phenol_CAS:57726-26-8 |  | 0,00434 | 0,00392 | 0,00190 | 0,00650 | 0,00343 | 0,00537 | 0,00821 | 0,00194 | 0,00291 | 0,00262 | 0,00271 | 0,00243 | 0,00438 | 0,00274 |
| Vanillin |  | 0,31287 | 0,21389 | 0,38876 | 0,37185 | 0,20260 | 0,36442 | 0,68103 | 0,33004 | 0,40699 | 0,38383 | 0,27980 | 0,23203 | 0,32590 | 0,38730 |
| Vanillyl_alcohol |  | 0,00204 | 0,00078 | 0,00078 | 0,00141 | 0,00081 | 0,00165 | 0,00296 | 0,00087 | 0,00171 | 0,00314 | 0,00066 | 0,00111 | 0,00150 | 0,00130 |
| p-Hydroxy-benzaldehyde |  | 0,00566 | 0,00309 | 0,00679 | 0,00502 | 0,00307 | 0,00550 | 0,01017 | 0,00541 | 0,00708 | 0,00852 | 0,00316 | 0,00415 | 0,00467 | 0,00575 |
