# Supplementary material for: Exploring the Potential of Lactic Acid Fermentation for the Recovery of Exhausted Vanilla Beans
Source: Front Nutr. 2022 May 19;9:858716. doi: 10.3389/fnut.2022.858716 (PMC9161551; doi:10.3389/fnut.2022.858716)

**Supplementary Figure 3** Volatile compounds recorded in fermented and unfermented samples of exhausted vanilla beans.. Concentrations are expressed in μg/g
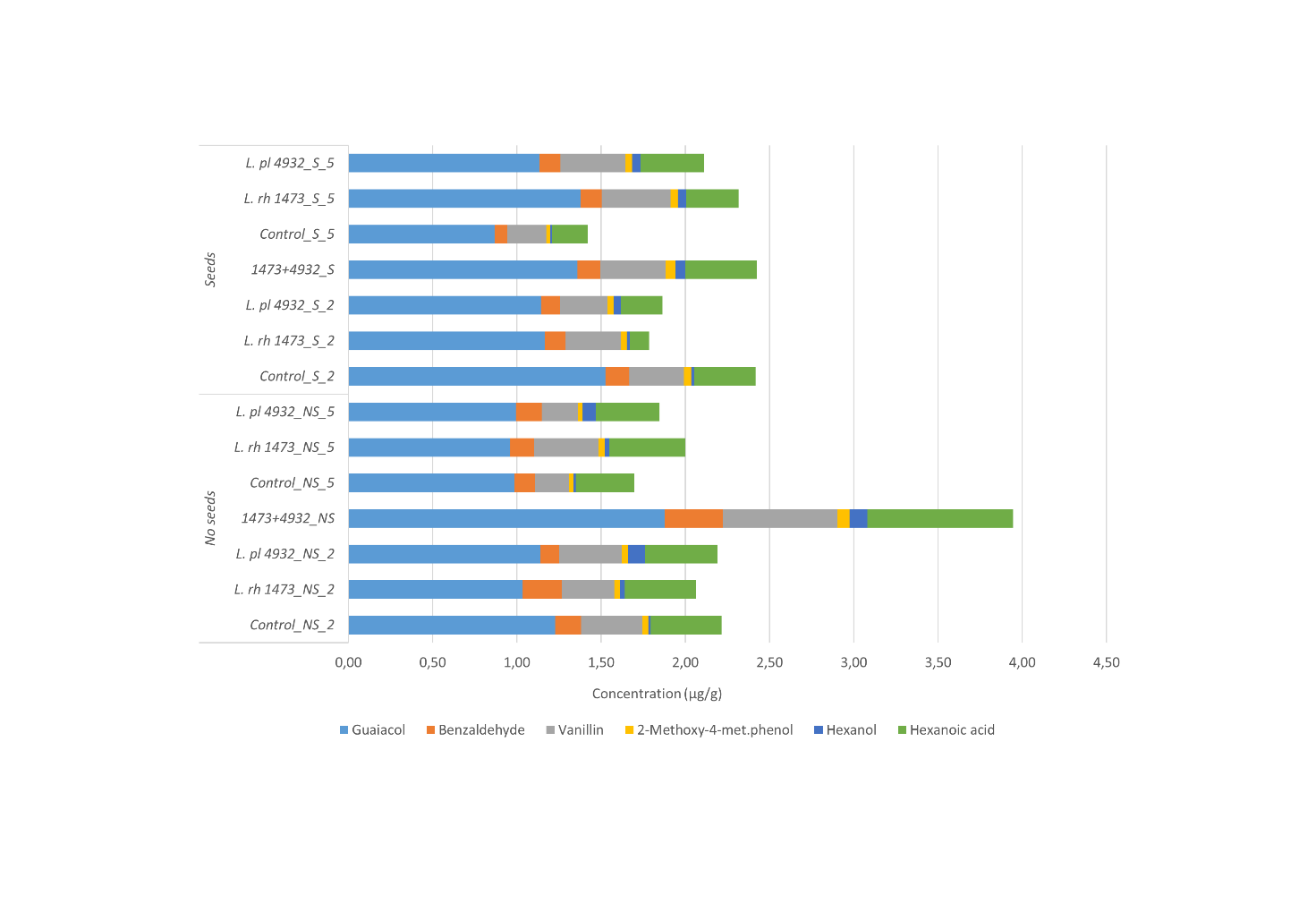

Supplement: Supplementary file 4 [file Table_4.DOCX]
